# *Drosophila* CASK regulates brain size and neuronal morphogenesis, providing a genetic model of postnatal microcephaly suitable for drug discovery

**DOI:** 10.1186/s13064-023-00174-y

**Published:** 2023-10-07

**Authors:** Judith A. Tello, Linan Jiang, Yitshak Zohar, Linda L. Restifo

**Affiliations:** 1 Graduate Interdisciplinary Program in Neuroscience, University of Arizona, Tucson, AZ 85721 USA; 2 Department of Neurology, University of Arizona Health Sciences, 1501 N. Campbell Ave, Tucson, AZ 85724-5023 USA; 3 Present address: Department of Molecular Pathobiology, College of Dentistry, New York University, New York, NY 10010 USA; 4 Department of Aerospace and Mechanical Engineering, University of Arizona, Tucson, AZ 85721 USA; 5 Department of Biomedical Engineering, University of Arizona, Tucson, AZ 85721 USA; 6 BIO5 Interdisciplinary Research Institute, University of Arizona, Tucson, AZ 85721 USA; 7 Department of Cellular & Molecular Medicine, University of Arizona Health Sciences, Tucson, AZ 85724 USA

**Keywords:** Intellectual disability, Haploinsufficiency, Short stature, Primary neuronal culture, Neurite arbor, Neurogenetics, Microfluidics, Immunostaining

## Abstract

**Background:**

*CASK*-related neurodevelopmental disorders are untreatable. Affected children show variable severity, with microcephaly, intellectual disability (ID), and short stature as common features. X-linked human *CASK* shows dosage sensitivity with haploinsufficiency in females. CASK protein has multiple domains, binding partners, and proposed functions at synapses and in the nucleus. Human and *Drosophila* CASK show high amino-acid-sequence similarity in all functional domains. Flies homozygous for a hypomorphic *CASK* mutation (*∆18*) have motor and cognitive deficits. A Drosophila genetic model of CASK-related disorders could have great scientific and translational value.

**Methods:**

We assessed the effects of *CASK* loss of function on morphological phenotypes in *Drosophila* using established genetic, histological, and primary neuronal culture approaches. NeuronMetrics software was used to quantify neurite-arbor morphology. Standard nonparametric statistics methods were supplemented by linear mixed effects modeling in some cases. Microfluidic devices of varied dimensions were fabricated and numerous fluid-flow parameters were used to induce oscillatory stress fields on CNS tissue. Dissociation into viable neurons and neurite outgrowth in vitro were assessed.

**Results:**

We demonstrated that *∆18* homozygous flies have small brains, small heads, and short bodies. When neurons from developing *CASK*-mutant CNS were cultured in vitro, they grew small neurite arbors with a distinctive, quantifiable “bushy” morphology that was significantly rescued by transgenic *CASK*^+^. As in humans, the bushy phenotype showed dosage-sensitive severity. To overcome the limitations of manual tissue trituration for neuronal culture, we optimized the design and operation of a microfluidic system for standardized, automated dissociation of CNS tissue into individual viable neurons. Neurons from *CASK*-mutant CNS dissociated in the microfluidic system recapitulate the bushy morphology. Moreover, for any given genotype, device-dissociated neurons grew larger arbors than did manually dissociated neurons. This automated dissociation method is also effective for rodent CNS.

**Conclusions:**

These biological and engineering advances set the stage for drug discovery using the *Drosophila* model of *CASK*-related disorders. The bushy phenotype provides a cell-based assay for compound screening. Nearly a dozen genes encoding CASK-binding proteins or transcriptional targets also have brain-development mutant phenotypes, including ID. Hence, drugs that improve *CASK* phenotypes might also benefit children with disorders due to mutant CASK partners.

**Supplementary Information:**

The online version contains supplementary material available at 10.1186/s13064-023-00174-y.

## Background

**Fig. 1:**
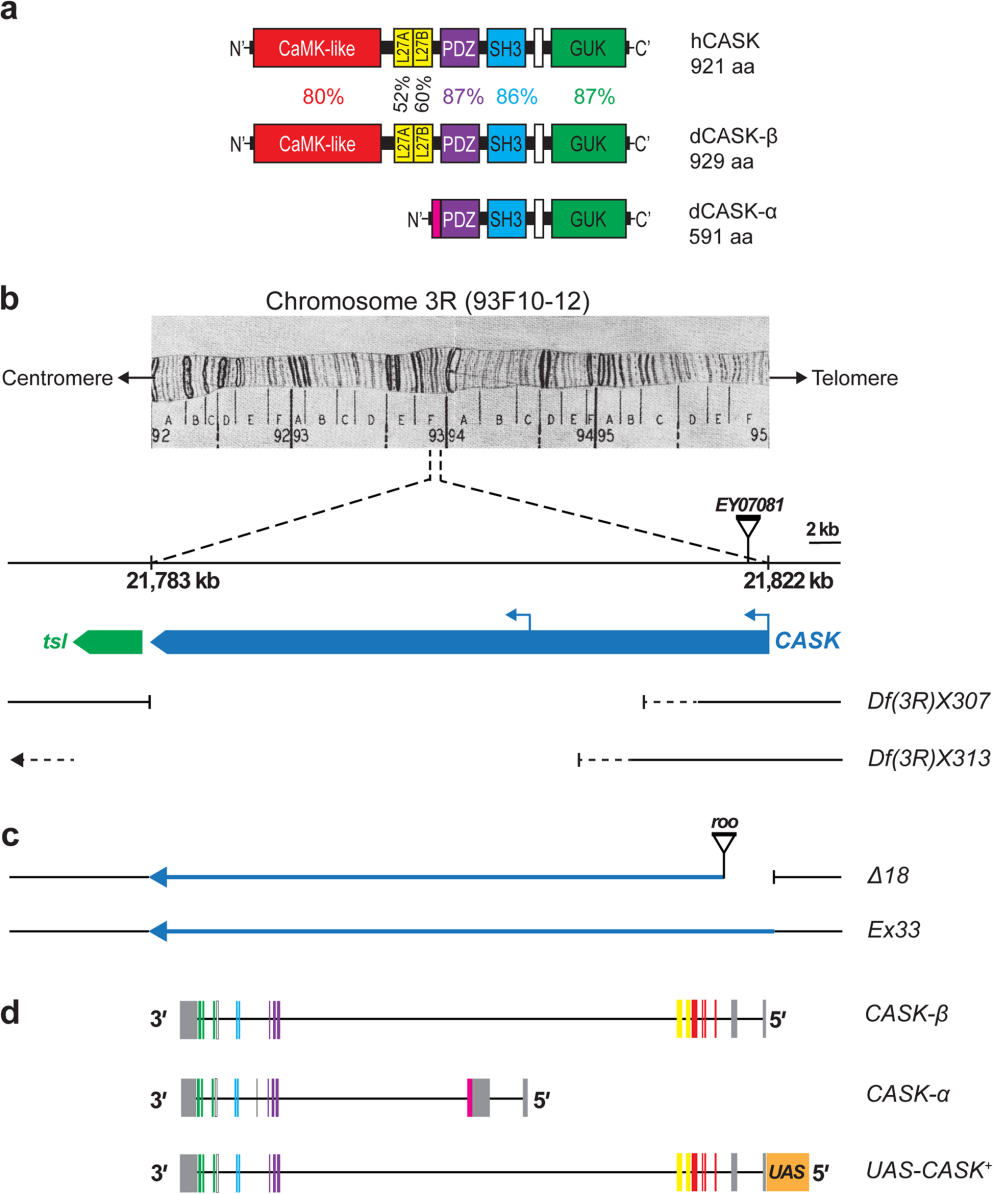
Drosophila *CASK* gene, proteins, and genetic reagents. **a** Color-coded schematic, drawn to scale, showing identical organization of CASK protein domains in *H. sapiens* and *D. melanogaster*. Amino-acid (aa) sequence similarity (%) as indicated; identities are CaMK-like 68%; L27A 31%; L27B 45%; PDZ 82%; SH3 67%; GUK 70%. The white boxes between SH3 and GUK are the "hook" motif. **b** Cytogenetic and molecular maps of *CASK* (FlyBase ID FBgn0013759; https://flybase.org/reports/FBgn0013759, last accessed 6 February 2023). Region 93F10-12 on right arm of chromosome 3 [86], reproduced in [91], centromere to the left. *CASK* gene span: genomic DNA map 21,783 – 21,822 kb (Fly Base, FB2022_06); scale at top right applies to all maps in (**b**)**-**(**d**). Protein orientation in (**a**) is flipped 180° relative to transcription direction. *CASK* (blue), with two transcriptional start sites (left-pointing arrows), and *tsl* (green) genes. P-element *EY07081* insertion site in first intron. Two overlapping chromosomal deficiencies delete *CASK* DNA, with breakpoints estimated by restriction mapping [96]. **c** *CASK* alleles [142]. Imprecise excision of *EY07081* generated *CASK* mutation, *∆18,* with deletion of exons 1 and 2, and insertion of small piece of *roo* transposon at the breakpoint. Precise excision yielded *Ex33*, which serves as the control allele. (**d**) Endogenous and engineered *CASK* transcripts. Boxes represent exons; color scheme for encoded protein domains as in (**a**), gray for 5'- and 3'-untranslated regions. *CASK-β* is the full-length transcript; internal promoter generates short transcript, *CASK*-α, which is missing the 5' exons. The *UAS-CASK*^+^ *10.20* construct expresses full-length wild-type *CASK* cDNA under UAS control

Originally identified as a neurexin-binding protein [53], CASK has numerous partners, each of which binds to one of its seven functional domains or motifs [106] (Fig. 1a, Additional file: Table A1). As more binding partners were discovered, it was proposed that CASK acts as a nucleation site, or scaffold, to aggregate proteins at specific subcellular sites [16]. In neurons, CASK localizes to both pre- and postsynaptic zones, as well as to the nucleus where it contributes to transcriptional regulation [59], Additional file: Table A1). CASK is regulated via phosphorylation by at least two kinases, CDK5 and PKA, which impacts its subcellular localization and function [62, 135]. CASK later emerged as essential for human brain development [102, 112].

Over 230 unique patients with mutations in the X-linked *CASK* gene (MIM# 300172; https://omim.org/entry/300172, last accessed 9 February 2023) have been reported, with five continents and many ancestries represented (Additional file: Table A2). Pathogenic loss-of-function (LOF) *CASK* mutations are found in all protein-coding domains [57]. Some mutant proteins had reduced binding to neurexin or other partners [79–81, 119, 151, 157]. Many genes encoding CASK transcriptional targets or partners also have neurodevelopmental mutant phenotypes (Additional file: Table A1). In network terms, *CASK* is the hub of a set of genes essential for brain development.

*CASK*-related neurodevelopmental disorders span a wide range of phenotypic severity. At the low end of the range is isolated or syndromic intellectual disability (ID), mostly but not exclusively in males and often accompanied by nystagmus (MIM# 300422; https://omim.org/entry/300422, last accessed 9 February 2023) [29, 34, 52, 124, 150]. Some of these patients have postnatal microcephaly. At the high end of the range is microcephaly with pontine-cerebellar hypoplasia (MICPCH, MIM# 300749; https://omim.org/entry/300749, last accessed 9 February 2023), a congenital disorder that causes severe psychomotor delay, often with early-onset, hard-to-control epilepsy, optic nerve/retinal abnormalities, and/or short stature. These patients have de novo germline *CASK* mutations, and most but not all are heterozygous females [47, 49, 55–57, 71, 80, 100, 103, 104, 112, 113, 120, 128, 129, 149, 153, 160]. In other cases, the phenotypes do not fit neatly into either category, including autism spectrum disorder (ASD) without ID in males or females [10, 64, 139], progressive microcephaly without cerebellar/pontine abnormalities [24], or focal cortical dysplasia in a female with a large somatic *CASK*-mutant clone [83]. With 41% of *CASK* clinical cases published in 2021 or later (Additional file: Table A2), many with novel mutations and phenotypes, it seems likely that the full phenotypic spectrum of *CASK*-related disorders remains to be revealed.

Several lines of evidence indicate that human brain development is very sensitive to CASK dosage and level of function. First, *CASK* shows haploinsufficiency, i.e., a single wild-type copy does not provide normal function in females. Females with ~ 50% of normal gene dosage due to heterozygous de novo germline whole-gene deletions, are most often born with MICPCH. Second, male infants with MICPCH are relatively rare, clinically very severe, dying as neonates or children [112, 120, 151]. Some surviving male patients with de novo mutations and MICPCH show somatic mosaicism [15, 57, 103], which would reduce the deleterious impact. The early lethality of the most severely affected males, along with the sex ratio of reported cases (F:M = 2.19, Additional file: Table A2) suggests that a significant fraction of males with germline de novo* CASK*-null mutations die either in utero or very young without a molecular diagnosis [103, 112]. In other words, *CASK* is likely an essential gene in humans, as it is in mice [8]. Third, weak *CASK*-LOF mutations, i.e., allowing considerable retention of function, cause X-linked recessive ID in males [10, 34, 52, 111, 124, 139, 150] with normal brain architecture (at MRI resolution) and variable features, including nystagmus, disruptive behavior, autism, constipation, facial dysmorphic, short stature, and microcephaly or relative macrocephaly. Those mutations may be inherited from a heterozygous or somatic-mosaic mother who was either very mildly affected or phenotypically normal [139, 151]. Taken together, the data are most consistent with MICPCH being X-linked semi-dominant, rather than dominant as classified by OMIM.

CASK protein-domain organization shows a high degree of phylogenetic conservation among animals, even with a simple nervous system [31, 82]. According to DIOPT v.9 (https://www.flyrnai.org/cgi-bin/DRSC_orthologs.pl, last accessed 14 June 2023), which integrates data from numerous orthology-prediction algorithms [61], *Drosophila melanogaster CASK* and human *CASK* are mutually orthologous. Reduction or absence of *Drosophila CASK* caused deficits of several types of associative learning or memory [48, 95]. In a striking example of conserved function, human *CASK*^+^ can rescue defective CaMKII autophosphorylation in *Drosophila CASK*-LOF mutants [48]. Several animal models of *CASK* disorders are available; each has distinct strengths and weaknesses vis-a-vis construct and face validity. Brain size was reduced in some mouse LOF models and recurrent seizures were seen in others [85, 145]. Reduced expression of zebrafish CASK caused microcephaly and provided an assay to assess functional changes of human mutations [24]. Here we report the novel findings that a partial-LOF allele of *Drosophila CASK* reduces the size of both brain and neurite arbors of cultured neurons.

Given the sensitivity of human brain development to CASK-function level, neonates with *CASK*-related disorders could benefit from treatments that boost CASK function or ameliorate downstream consequences of the mutation. A needed step toward that goal is assay development for drug discovery. Using a partial-LOF *CASK* mutation, we developed a *Drosophila* model of mild-to-moderate *CASK*-related disorders with microencephaly that also provides a cell-based assay for phenotypic analysis and screening. Moreover, the network relationships between CASK and genes encoding its partners (Additional file: Table A1) suggest therapeutic strategies for a whole set of monogenic disorders.

In the past decade, microfluidic systems and technology have advanced to enable single-cell neuronal analyses [51] and drug screening using a variety of cell-based assays [28, 166]. A major bottleneck remains the dissociation of viable neurons from brain tissue. We developed microfluidic technology for standardized, automated brain tissue dissociation in which the *CASK*-mutant neuronal phenotype is retained with high fidelity. These advances will enable compound screening of primary cultured neurons as a stepping stone toward therapeutic development.

## Methods

### Amino acid sequence comparison

We compared *Homo sapiens* CASK isoform 1 (NCBI Reference Sequence NP_003679.2) to *Drosophila melanogaster* CASK isoform H (NCBI Reference Sequence NP_001262811.1) using the BLOSUM62 similarity matrix [58] and the EMBL-European Bioinformatics Institute (EBI) tools collection (https://www.ebi.ac.uk/Tools/, last accessed 25 February 2022). The conserved protein-domain boundaries were based on HMMER's HMMSCAN and their pairwise sequence identities and similarities determined by EMBOSS program Needle. The dot-matrix display of amino acid similarity was generated by EMBOSS program Dotmatcher [94]) using a Needleman-Wunsch global alignment. The settings selected were window length of 10 amino acids and score threshold of 30, which is somewhat more stringent than the default threshold of 23.

### *Drosophila melanogaster* genetic reagents and crosses

Fly stocks were maintained at room temperature (21–23 °C) on a masa corn flour-nutritional yeast-agar medium [37] supplemented with active baker’s yeast. Cultures for experiments were reared at optimal larval density under standard conditions of 25 °C, 60–80% relative humidity, as described previously [74]. Stocks of X-ray-induced third-chromosome deficiencies, *Df(3R)X307* and *Df(3R)X313* (FlyBase ID FBal0048070 and FBal0048071, respectively; Fig. 1b), were obtained from the Bloomington *Drosophila* Stock Center (Bloomington, IN), and maintained over the balancer *TM6B, Tb e.* Based on low-resolution mapping [96], *Df(3R)X307* is the smaller deletion with breakpoints within the gene, removing most of *CASK*. *Df(3R)X313*, deletes most of *CASK* and additional downstream sequences, with an unknown left-hand breakpoint. Each deficiency is homozygous lethal during embryonic development, but neither recessive lethal has been mapped.

Three *CASK* stocks (Fig. 1c, d) were gifts from Dr. Leslie Griffith (Brandeis University, Waltham, MA): (i) the hypomorphic allele, *∆18* (also known as *P18*), (ii) a precise-excision control allele, *Ex33* (also known as *Con*; [142], and (iii) a second-chromosome-insertion line of *UAS-CASK*^+^ *10.20* [32] generated in the laboratory of Dr. Peter Bryant (University of California—Irvine). *∆18* was derived by imprecision excision of P-element EY07081 (Fig. 1b) from the first intron of *CASK*, 1751 bp upstream of the translational start site in exon 2 of the full-length transcript [142]. This excision event caused a 2624-bp deletion, including exons 1 and 2, as well as insertion of a small piece of *roo* transposon at the breakpoint. Thus, full-length CASK is absent in *∆18* homozygotes, while the smaller CASK-α can be produced from the internal promoter [142], Fig. 1b-d).

A second-chromosome reporter line expressing a modified GFP with greater stability under GAL4 control, *UAS-GFP-S65T*, was engineered by Dr. Barry Dickson (then at University of Zürich) and obtained from Dr. Kei Ito (then at Japan Science and Technology Corporation, Tokyo; [65]. A stock with the *Gal4* driver inserted in the X-linked *elav* gene [89], *elav-Gal4*^*C1555*^*; CyO/Sp; Sb/TM3, Ser*, was provided by Dr. Konrad Zinsmaier (University of Arizona, Tucson, AZ). The *elav-GAL4*^*C155*^ driver is often referred to as “pan-neuronal” because *elav* is expressed in all neurons in vivo [89, 131, 144]. However, the *C155* line had never been evaluated in primary neuronal culture. In neuronal cultures from the central nervous system (CNS) of female larvae with one copy each of *elav-GAL4*^*C155*^ and *UAS-GFP-S65T*, we found that ~ 85% of neurons were GFP-positive, whether visualized live or after immunostaining for GFP (data not shown). Among GFP( +) neurons, there was no obvious variation in labeling intensity. Therefore, in the context of primary neuronal cultures from larval CNS, *elav-GAL4*^*C155*^ is not fully pan-neuronal, but rather drives expression in a large majority of neurons. A *repo-lacZ* reporter line, *ry*^*506*^* P{PZ}repo03702/TM3, ry*^*RK*^* Sb*^*1*^* Ser*^*1*^, obtained from the Bloomington *Drosophila* Stock Center, was used to detect glia in dissociated CNS.

To generate *CASK* heterozygotes (*Δ18 / Ex33*)*, w/w;* + */* + *; Δ18 / Δ18* virgin females were crossed with *w/Y;* + */* + *; Ex33/ Ex33* males. For deficiency analyses with *Df(3R)X307* and *Df(3R)X313*, *Df(3R)/TM6B, Tb e* flies were crossed in parallel with homozygous *Ex33* and *∆18* flies to yield hemizygous control, *Ex33/Df(3R),* and hemizygous mutant, *∆18/Df(3R)*, progeny. To assess transgenic rescue, we used GAL4-UAS [13] to control expression of *CASK*^+^ in a *CASK*-mutant background. *UAS-CASK*^+^ *10.20* encodes a wild-type cDNA that corresponds to full-length *CASK-β* (Fig. 1d), *CASK-RB* mRNA (FlyBase CG6703-RB), expressed under GAL4 control [32]. The neuron-specific driver *elav-Gal4*^*C155*^ and the *UAS-CASK*^+^ transgene were individually crossed into the *CASK*-mutant background (*∆18/ ∆18*) and then crossed to each other:


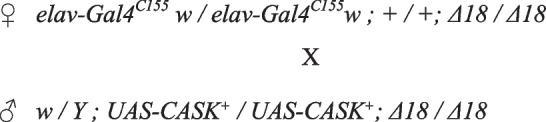


Female progeny, *elav-Gal4*^*C155*^* w / w; UAS-CASK*^+^*/* + *; ∆18 / ∆18*, were selected as wandering third instar larvae (w3L) for neuron-culture experiments.

### Quantitation of body, head, and brain sizes

#### Measurement and microdissection

**Fig. 2:**
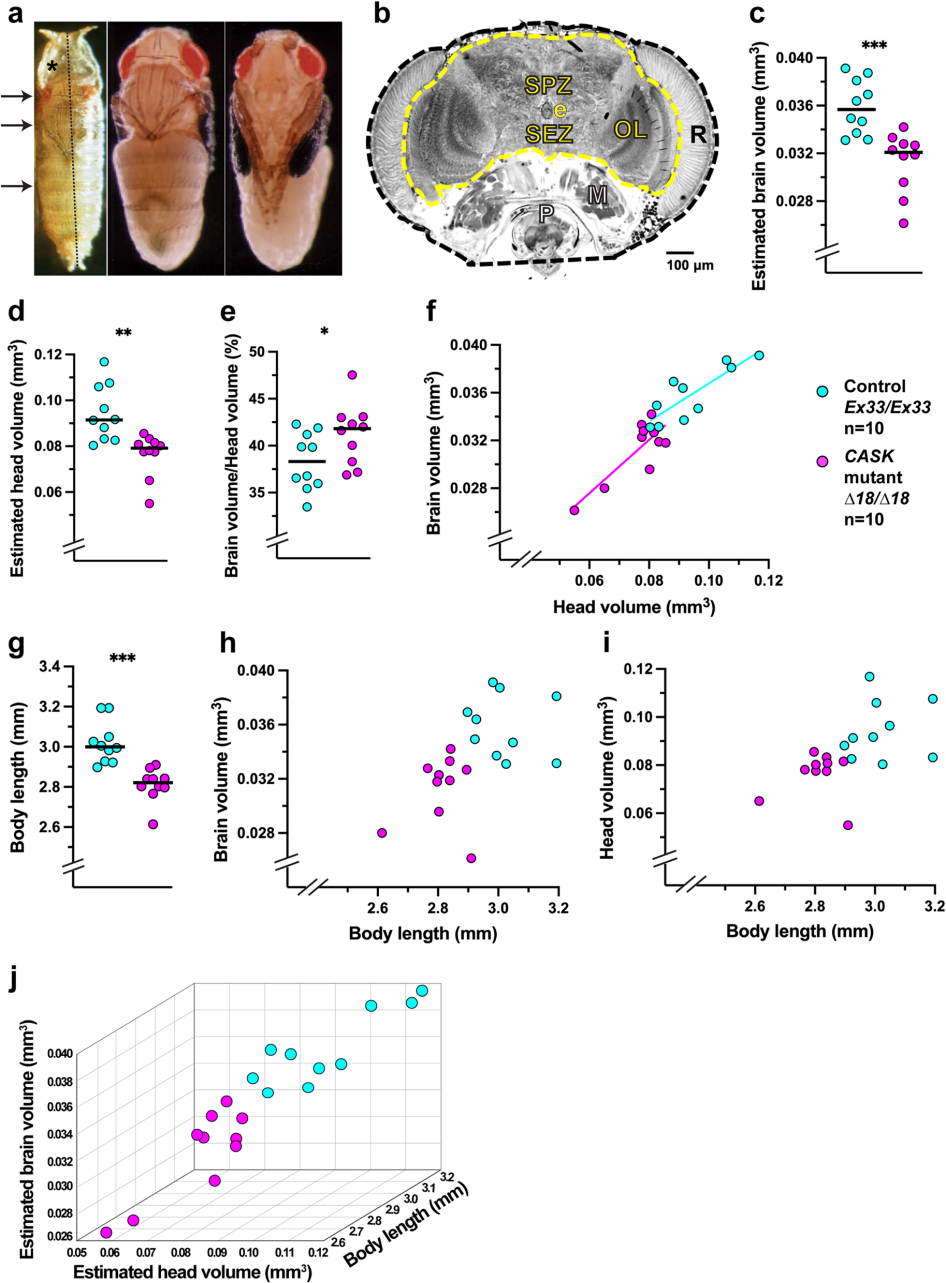
The morphological phenotype of *CASK* mutants involves head, brain, and body. **a** Photomicrographs of *Drosophila* pharate adults, anterior at the top. Left, Canton-S wildtype within the puparium (also called "pupal case"), dorsal view. Vertical dotted line represents body length. Arrows show the centers of the head, thorax, and abdomen (top to bottom, respectively). Asterisk marks the air space anterior to the animal. Center and right, dorsal and ventral views, respectively, of a female genetic control, dissected from the puparium but still wrapped by the transparent pupal cuticle. Adapted from [41], with permission. **b** Photomicrograph of a histological section through the head of a *CASK*-control (*Ex13/Ex13*) pharate adult, frontal plane. Scale bar as indicated. Colorized and thickened dashed outlines overlay the perimeters of the CNS (yellow) and head (dark blue), traced using cellSens software. e, esophagus; M, muscles; OL, optic lobe; P, proboscis; R, retina; SEZ, subesophageal zone; SPZ, supraesophageal zone. (c-j) Abnormal morphology of *CASK Δ18/Δ18* mutants compared with *Ex33/Ex13* controls. Each circle represents a single fly; color legend shown to the right of panel f. Horizontal black lines are medians. Significance levels: *, *p* < 0.05; **, *p* < 0.005; ***, *p* < 0.0005. In *CASK* mutants, **c** estimated brain volume was reduced (*p* = 0.0002); **d** estimated head volume was reduced (*p* = 0.0007); **e** the percentage of head volume occupied by the brain was increased (*p* = 0.0288); **f** scatter plot of head volume vs. brain volume shows a positive linear relationship in both mutants and controls; (**g**) body length was reduced (*p* < 0.0001); **h**-**i** scatter plots of body length vs. brain volume or head volume show distinct distributions of mutant and control data and no strong indication of linear relationships; **j** in a 3D scatter plot of brain volume, head volume, and body length, the data from the two genotypes are nonoverlapping, which was confirmed by rotating the graph

Males of known genotype at the P14 late pupal stage [9] were individually positioned dorsal side up on a glass microscope slide. Digital images were captured by a Canon Rebel T3i EOS 600D digital SLR camera, with a Canon EF 100 mm f/2.8 Macro USM lens (Canon USA, Inc., Melville, NY), mounted on a tripod to standardize the distance between the camera and the lab bench. In each image, the length of the puparium (often called “pupal case”) was measured along the midline between the anterior and posterior spiracles (Fig. 2a) using the ImageJ tool ‘Measuring Distance Between Points’ [137]. A stage micrometer with 0.01 mm subdivisions (Ward's Science, Rochester, NY) provided the scale. Each pupa was transferred to PBS, pH 7.4, in a glass dissecting well. The puparium was opened at the anterior end. The pupal cuticle, a transparent, thin membrane that shrink-wraps the developing fly, was removed from around the head. Using one tip of fine forceps as a blade (Dumont #5, Dumoxel with Biologie tips; Fine Science Tools, Inc., Foster City, CA, USA), the head was severed from the body and the distal proboscis removed. The latter step provides an entry point for reagents, enhancing fixation and embedding of internal soft tissues.

#### Fixation and embedding

Each head was processed individually. The protocol was modified from one used for estimating brain volumes of larger insects [130]. Unless otherwise stated, chemicals were purchased from Electron Microscopy Sciences (Hatfield, PA, USA). Heads were fixed overnight in freshly diluted 4% formaldehyde in PBS, pH 7.4, at 4 °C on a platform rocker (Roto-Shake Genie, Scientific Industries, Bohemia, NY, USA). Unless otherwise stated, all subsequent steps were at room temperature. After fixation, each head was transferred to a microcentrifuge tube and washed twice in PBS, pH 7.4, for 1 h each, and in distilled water for 1 h. Washes and subsequent steps were done on the platform rocker. To stain the tissue, the heads were incubated in osmium tetroxide, 1% in H_2_O, for 2 h at 4 °C and an additional hour at room temperature, then rinsed in water for 3 h. A one-step dehydration method [23, 36] was performed by placing samples in 2,2-dimethoxypropane (TCI America, Portland OR, USA), acidified to ~ 0.03% v/v hydrochloric acid, for 15 min. After dehydration, heads were first immersed in 100% acetone for 20 min, then transferred to 30% acetone/70% Spurr low-viscosity embedding resin for 6 h, and finally into 100% resin overnight. Each specimen was mounted in a BEEM™ (VWR) embedding capsule filled with 100% Spurr resin, and oriented for sectioning in the frontal plane; occasionally, the head would tilt before the resin solidified. Resin polymerization was induced by overnight incubation at 70 °C.

#### Serial sectioning

Each polymerized block was secured in a specimen holder and trimmed into a rectangle around the head using a disposable single-edge razor blade. Serial sections, 12 µm, were cut on a sliding microtome (Reichert, Austria) with a stainless-steel knife. Serial sections of each head were arranged using a moist paintbrush in rows on a single microscope slide on a warming tray at 60 °C to facilitate flattening. Section recovery was > 99%; lost sections were documented. The slides were mounted with Cytoseal (Thermo Scientific™, Waltham, MA) and coverslipped. These are permanent preparations.

#### Image acquisition and analysis

Digital images of each histological section (Fig. 2b) were acquired with an Olympus DP70® camera mounted on an Olympus BX51TF® (Tokyo, Japan) compound microscope, using a 20X objective (numerical aperture, 0.50). For any section that was not flat, several images were acquired at different focal planes, merged using Adobe Photoshop (Adobe Systems Inc., San Jose, CA), and adjusted for brightness and contrast. To estimate volumes from serial sections, Olympus cellSens™ Entry software (v.1.14) was used to quantify the area of anatomical structures. Outlines of the head perimeter and of the CNS therein (“brain”) were traced using the *measure polygon* tool, which provides the number of pixels within the outlined area. To approximate the volume contributed by the proboscis, the ventral margin of the head was drawn across the midline, connecting the right and left ventral-most points (Fig. 2b). In sections with non-contiguous CNS tissue, several polygons were outlined and measured. A stage micrometer (Ward's Science, Rochester, NY, USA) was used for calibration. In the case of one lost section, the values of its head and brain areas were assumed to be midway between those of the flanking sections. Several sections (< 0.5%) were partly folded, obscuring one side of the head/brain; this was estimated from the area of the contralateral side. The summed areas of all sections of each sample were multiplied by section thickness (12 µm) to estimate head and brain volumes.

### Primary neuronal cultures from developing CNS tissue

Dissociated neuronal cultures were prepared from the whole CNS of w3L as previously described [74, 75, 143]. At this stage of development, CNS cells include mature neurons, incompletely differentiated neurons, glia, and neural stem cells. For comparison among genotypes, larvae were matched for sex and culture density. Cell-culture medium was prepared using two fetal bovine serum (FBS) sources at different times. Most experiments used FBS from Hyclone (Logan, UT; lots APC20860 or AZA180873), while a few used Optima FBS (Atlanta, GA; lot #D0118); Hyclone FBS supported better neurite outgrowth. After incubation in enzymes that digest the extracellular matrix, the CNS was dissociated either by manual trituration [143] or in a microfluidic device (see below). Both methods generate suspensions of viable neurons and neural stem cells. Dissociation severs all axonal and dendritic extensions from neurons, which re-elaborate arbors during in vitro culture. Neural stem cells proliferate for at least one day in vitro (div; data not shown); their progeny neurons then differentiate de novo.

Each CNS-cell suspension was plated by distributing it into five or six custom culture dishes [143], either made in-house or purchased from MatTek (Ashland, MA). After 2–3 h at 25 °C to allow the cells to adhere to the substrate, the dishes were flooded with culture medium and maintained at 25 °C for 3 div (~ 70–80 h post-plating). Neurite outgrowth starts within hours, and a complex neurite arbor can be seen at 3 div [74, 143]. Overlap between neighboring arbors is usually limited, allowing evaluation of individual neurons. Data acquisition and analyses were 'blinded' by having dishes coded for preparation method and genotype by a lab member not involved with that experiment.

Rat and mouse E18 hippocampi were purchased from BrainBits® (Springfield, IL, USA; now Transnetyx Tissue) which ships tissue in a proprietary physiological stabilization solution, Hibernate® EB (HEB), along with supplies for neuronal culture. Each hippocampus was cut into four pieces with iridectomy scissors. The pieces were transferred in a sterile 9″ Pasteur pipette with minimal HEB medium into freshly-made papain (2 mg/ml in Hibernate® E without calcium or B27®). The tissue was digested for 10 min at 30 °C, with gently swirling every 2 min. Following digestion, the tissue was transferred to fresh HEB at room temperature for 5–10 min. prior to loading each piece into a microfluidic device for dissociation. The recovered cells were sedimented by brief centrifugation, washed in NbActiv1® plus glutamate (Neurobasal® + B27® + Glutamax®), and plated on glass coverslips coated with poly-D-lysine. The cells were flooded with the same medium and cultured for 4–5 div at 37 °C with 5% v/v CO_2_.

### Neurite-arbor size and shape analysis

Fixation for immunostaining of cultured neurons was performed as described previously [74, 76, 143]. The neuronal membrane protein Nervana 2 [146] was labeled with polyclonal goat anti-horseradish peroxidase (anti-HRP) (Sigma, St. Louis, MO) at 1:500, detected by Donkey anti-Goat IgG Alexa Fluor® 488 or Donkey anti-Goat IgG Alexa Fluor® 568, both from Molecular Probes (Life Technologies), diluted 1:500. Nervana 2's high abundance and uniform distribution provide a strong, contiguous signal along the neurite as it tapers to a fine tip. Stained cultures were mounted under a coverslip in 12% w/v polyvinyl alcohol (Sigma, St. Louis, MO) with 1.5% w/v DABCO (1,4-diazabicyclo [2.2.2] octane; Sigma), an anti-fading reagent, and stored at 4 °C in the dark.

Neuronal nuclei were identified with anti-ELAV mouse monoclonal antibody, mAb5D3C5 [132, 144] at 1:100, detected by Goat anti-Mouse Cy3 (Jackson ImmunoResearch, West Grove, PA, USA) at 1:400. To identify glia expressing the *repo-lacZ* reporter, we used pre-absorbed rabbit polyclonal anti-β-galactosidase (β gal) (Cappel, Malvern, PA, USA) at 1:50 as described [76] and Goat anti-Rabbit Alexa Fluor® 488 (Molecular Probes, Life Technologies) at 1:300. This strategy allowed neurons and glia to be labeled in the same cultures. To detect GAL4-driven GFP expression by immunostaining, chicken anti-GFP IgG (Life Technologies) was used with Goat anti-Chicken IgG Alexa Fluor® 488 (Molecular Probes), both at 1:500.

Live and immunostained cultured *Drosophila* neurons were examined on a Diaphot 300 inverted microscope (Nikon, Tokyo, Japan), with 40X (numerical aperture, 1.0) or 60X oil-immersion (numerical aperture, 1.4) objectives, using phase-contrast and fluorescence optics. Fluorescent signal was detected with filter cube Chroma #41001 (exciter 460–500 nm; dichroic 505 nm; bandpass emitter, 510–560 nm) for Alexa Fluor® 488 and with Nikon G-2A filter cube (exciter, 510–560 nm; dichroic 580 nm; long-pass emitter, 590 nm) for Alexa Fluor® 568 and Cy3. Images were acquired with Hamamatsu ORCA-285 digital camera (Hamamatsu Photonic Systems, Bridgewater, NJ, USA) and HCImage® software (v.2.0.0.0; Hamamatsu). Hippocampal neurons were viewed at 10X, 20X, or 40X under phase-contrast.

For quantitative analysis of neurite arbors, a systematic sample of ~ 100 neurons (i.e., cell body and complete neurite arbor) was imaged from a single culture dish, using the staircase-sampling method [143]. Neurons with modest overlap of neighboring arbors were also included. Exclusion criteria for imaging a neuron included weak anti-HRP signal (suggesting cell death), damaged cell bodies, and overlapping arbors that could not be resolved to allow accurate image analysis. For neurons with larger arbors extending beyond the 60X microscopic field, multiple overlapping images were acquired and merged by “stitching”, using PanaVue ImageAssembler® software (Panavue Inc., Québec, Canada).

Digital images were converted from 16-bit to 8-bit tiff for analysis by semi-automated custom software, NeuronMetrics™ [115, 143], which converts each neurite arbor into a one-pixel-wide skeleton connected to the cell body and bounded by the neuron's convex polygon (“territory”). Two key features of NeuronMetrics™ are algorithms that (i) connect gaps in the skeleton (due to variation in fluorescence intensity) and (ii) correct branch numbers for neurite-to-neurite contacts within the same arbor. NeuronMetrics™ computes number of primary processes (also called primary neurites), neurite-arbor total neurite length, branch-number estimate, territory area, and territory perimeter. From these, additional measures were calculated: higher-order branch number (total branches minus primary neurites), branch density (branches per 1000 µm^2^ territory area), and branches per unit length. After statistical analysis, the code was broken.

### Microfluidic systems for brain-tissue dissociation

The microfluidic system includes several components: (1) a transparent microdevice with one or more parallel channels; (2) an external fluid-handling system (including a programmable syringe pump, a pressure transducer, a valve, inlet/outlet connectors, and tubing); (3) a microscope and camera for real-time monitoring and image acquisition; and (4) a computer connected to both the pump and microscope. Microdevice design and fabrication were similar to those previously described [68] with numerous design variations tested for optimization. Single- and dual-microchannel devices were fabricated from molded polydimethylsiloxane (PDMS; Sylgard 184 silicon elastomer, Dow Corning, USA) using standard microfabrication techniques. In brief, a computer numerical control (CNC) machine was used to create reusable aluminum molds with the designed microchannel patterns. A mixture of PDMS base and curing agent was poured into the mold(s) and polymerized at 50 °C. After peeling off the cured PDMS sheet from the mold, holes were punched at each end of the channel groove for the inlet/outlet ports. The device fabrication process was completed by bonding the molded PDMS substrate to a flat PDMS substrate following oxygen plasma treatment. The inner surfaces of the formed PDMS channel were highly hydrophobic preventing adhesion of tissue or neurons during experimentation. These methods are well-established to be biocompatible [141].

The overall channel length was ~ 40 mm with a mirror-image symmetric design. Each channel was 2 mm wide at its distal ends, gradually tapering to 1 mm approaching the center. Tissue dissociation, driven by oscillatory fluid flow, took place in the narrow central orifice flanked by the two wider segments. The side walls in the transition zone between the wider channel segments and the orifice form ~ 45-degree angles symmetric with respect to the channel central axis. For sterilization, the channels were flushed with 70% ethanol prior to connecting them to the pump, followed by device treatment under UV light. Because PDMS is elastic, the device was sandwiched between two rigid acrylic plates during the dissociation experiment to keep the orifice dimensions fixed under high pressure.

Fluid flow was driven by an Infuse/Withdraw PHD ULTRA syringe pump (Harvard Apparatus, Holliston, MA, USA) controlled by a custom-designed interface in LabVIEW (National Instruments, 2015, USA) running on a standard desktop computer with Windows operating system. The tubing connecting the pump to the microdevice had internal diameter 1/16 inch. The system was primed with fresh culture medium prior to sample loading. Enzyme-treated brain tissue (fruit fly or rodent) in fresh culture medium was loaded manually with a micropipette (Gilson, Middleton, WI, USA) and positioned adjacent to the central orifice under direct microscopic observation. Numerous combinations of fluid-flow parameters (infusion volume, oscillation frequency, flow rate and cycle number) were tested for each tissue type, with most assessments by holistic scoring (excellent, very good, good, fair, poor).

### Statistics and graphing

Pair-wise comparisons of individual parameters between genotypes were made using the non-parametric Mann–Whitney rank sum test for non-normally-distributed data [40]. The tests were implemented in RStudio (RStudio 2022.07.2 + 576 "Spotted Wakerobin" Release,R version 4.2.2 GUI 1.79 High Sierra build; R Core Team, 2022) under the GNU General Public License, using scripts developed by J.A.T. Graphs were created using R scripts or GraphPad Prism (version 9.0.0 for Mac, GraphPad Software, San Diego, CA, USA). Photomicrographs of immunostained neurons underwent cropping and "Autocontrast" adjustment in Adobe Photoshop. Figures were assembled and edited using Adobe Illustrator version 26.4.1 (Adobe, Inc., San Jose, CA, USA).

Comparisons of *∆18* and *Ex33* homozygotes performed over several years were analyzed using a linear mixed effects model. This allows use of data across multiple repeated studies with inter-experiment variability, e.g., due to differences in reagents and environments. For each neurite-arbor parameter, each experiment represents a single sample with a summary statistic, the median. The method first calculates the genotype-to-genotype median difference within each experiment, the mean of the median differences across all experiments, standard error of the mean, and 95% confidence interval. The mixed effects model fits a random intercept for each experiment (medians) and genotype fixed effects within an experiment. For each parameter, the differences between genotypes are subjected to a two-tailed t test. The analysis was implemented in the R package nlme using the lme function (version 3.1–160; [127]).

## Results

### A *Drosophila CASK*-LOF mutation causes microcephaly, microencephaly, and a form of short stature

We found that, despite 700 million years of evolutionary distance from a common ancestor, human and fruit fly CASK show remarkable phylogenetic conservation (Fig. 1a; Additional file: Fig. A1^4^). The functional domains of *Drosophila* CASK have 52–87% amino-acid similarity to those of human CASK, with the highest in the PDZ and GUK domains (87% for both) and lowest in the L27 domains. Human *CASK* mutations cause microcephaly and short stature, which co-occur in a subset of monogenic neurodevelopmental disorders with intellectual disability [14]. We used the partial-LOF *CASK* mutation (Fig. 1c) to determine whether *Drosophila CASK* controls brain size and body length, comparing *Δ18* homozygous mutants with *Ex33* homozygous controls.

*CASK*-mutant *∆18* homozygous flies do not have obvious malformations of either head or brain development. For brain- and head-volume estimates, we analyzed serial histological sections of fly heads at the end of metamorphosis (Fig. 2b), when brain morphology is mature at the light-microscopic level. We are using "brain" to mean all CNS tissue in the head – the cerebral and gnathal ganglia, also called supra- and subesophageal zones [66],this includes structures functionally analogous to the brain and brainstem of mammals. *CASK*-mutant flies had significantly smaller brains compared to genetic controls (*p* < 0.001; Fig. 2c). Thus, the *CASK*-mutants have microencephaly. *CASK* mutants also have smaller heads than their genetic controls (*p* < 0.001; Fig. 2d); in other words, *CASK*-LOF mutants have microcephaly. This was somewhat surprising because, while mammalian head growth is mechanically driven by brain growth, the two processes are anatomically separate during *Drosophila* development [43]. Quantification of the percentage of head volume occupied by the brain, showed that *CASK*-mutant brains occupied a slightly but significantly greater fraction of the head (*p* < 0.05) compared to genetic controls (Fig. 2e). This suggests that the reduction in head size of *CASK*-mutant flies is somewhat more severe than their reduced brain size. The positive linear relationship between brain and head volumes (Fig. 2f) seen in controls is retained in the mutants, with a shift in data distribution to lower values but no significant difference in slopes derived from linear regression analysis. (Controls: y = 0.159x + 0.021; R^2^ = 0.71. Mutants: y = 0.224x + 0.014; R^2^ = 0.67).

We measured body length (head-thorax-abdomen) along the longitudinal axis of the protective cocoon-like puparium (Fig. 2a) which forms from the larval cuticle at the start of metamorphosis. It directly reflects larval length, which dictates the length of the adult. It is stable, stationery, and firm, hence easy to measure. In comparative anatomy terms, insect-puparium length is most similar to mammalian crown-rump length (CRL), a commonly used measure of fetal growth in humans and other vertebrates [77, 123]. *CASK Δ18/Δ18* mutants have significantly shorter body length than *CASK Ex33/Ex33* controls (*p* < 0.0005) (Fig. 2g). Thus, like the human orthologue, *Drosophila CASK* promotes growth along the longitudinal body axis. This is also consistent with an average ~ 15% reduction in body weight of *∆18/∆18* compared with *Ex33/Ex33* [42].

When brain and head volumes were compared with body length, the relationships do not appear linear for either genotype (Figs. 2h, i, respectively). This suggests that the reduced brain and head volumes of *CASK* mutants are not simply a consequence of reduced animal size. In a 3D scatter plot of body length, head volume, and brain volume (Fig. 2j), the data from mutants and controls form two non-overlapping groups. In summary, *Drosophila CASK* is required for the developmental attainment of normal brain volume, head volume, and body length.

### *CASK* LOF disrupts neurite-arbor morphogenesis: the bushy phenotype

**Fig. 3:**
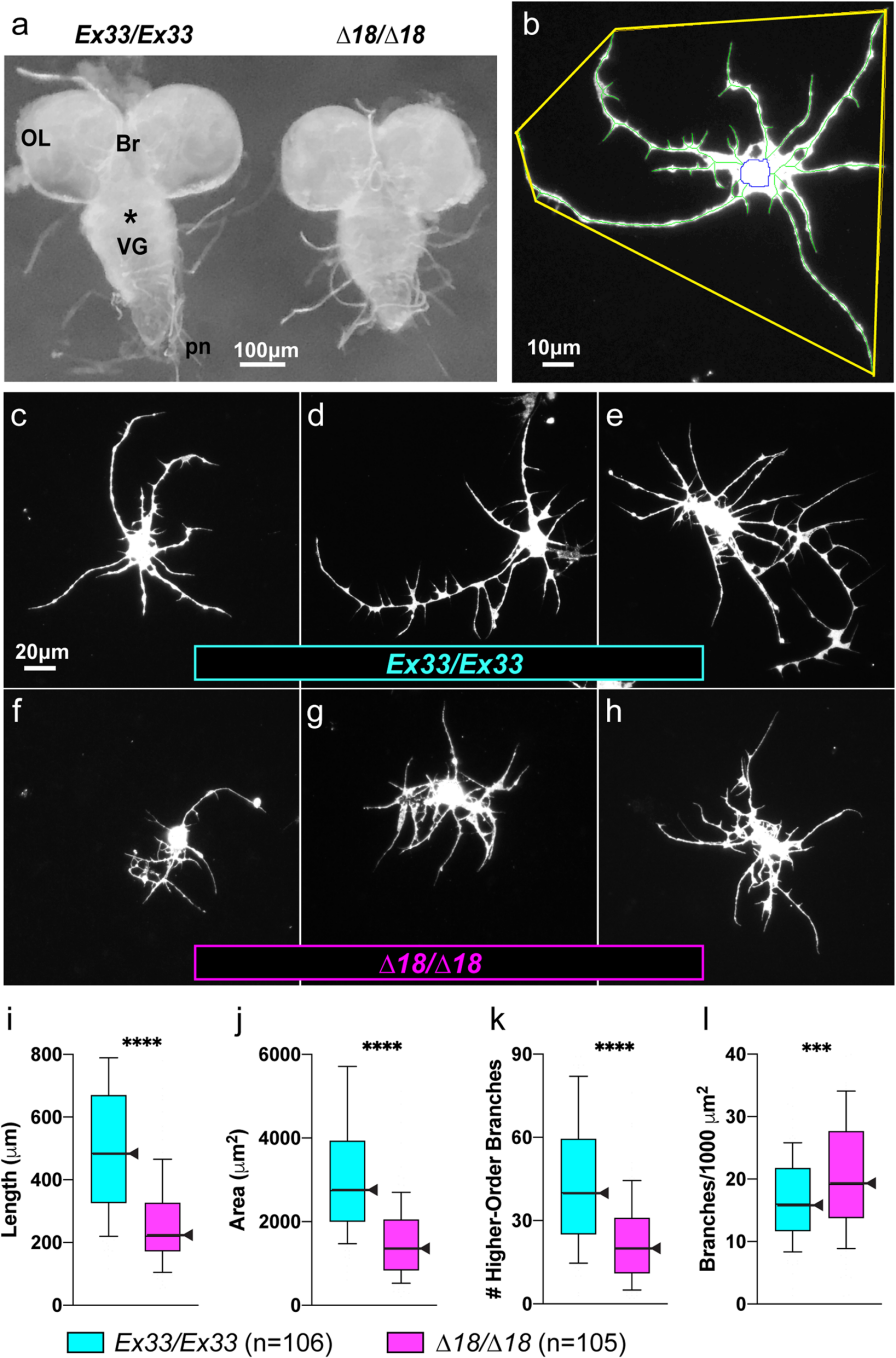
The “bushy” phenotype of *CASK-*mutant neurite arbors in vitro. **a** The *CASK-*mutant larval CNS is reduced in size. Stereomicroscopic view of freshly dissected, unfixed whole-CNS explants from wandering third-instar female larvae in buffered saline, dorsal side up, anterior to the top. *CASK* control (*Ex33/Ex33*; left) alongside mutant (*∆18/∆18*; right). OL, optic lobe; Br, brain; *, subesophageal zone; VG, ventral ganglia; pn, peripheral nerves. Scale bar = 100 µm. **b**-**h** Photomicrographs of cultured larval CNS neuron, immunofluorescently labeled for a neuronal membrane marker, after 3 div (60X magnification). **b** NeuronMetrics software output. The color-coded lines have been thickened to improve visibility: yellow polygon, territory; blue, central portion of the neuronal cell body; green, branches of the skeletonized neurite arbor. Scale bar = 10 µm. **c**-**h** Scale bar = 20 μm. Neurons representing the ~ 25^th^ (**c**, **f**), ~ 50^th^ (**d**, **g**), and ~ 75^th^ (**e**, **h**) percentiles for each of three arbor-size parameters, total neurite length, territory area, and branch density. **c**-**e** *CASK* control, *Ex33/Ex33*. **f**–**h** *CASK* mutant, *∆18/∆18*. (**i**-**l**) The “bushy” phenotype: *CASK*-mutant neurite arbors are reduced in size with excessive branch density. Quantification of neurite-arbor morphology, depicted as box-plot distributions, comparing 3 div cultures of *CASK*-control (*Ex33/Ex33*; *n* = 106 neurons; aqua) and *CASK-*mutant (*Δ18/∆18*; *n* = 105 neurons; magenta) larval CNS neurons. Center lines and arrowheads represent the 50^th^ percentile. Top and bottom of each box represent the 75^th^ and 25^th^ percentiles, respectively. The upper and lower whiskers represent the 90^th^ and 10^th^ percentiles, respectively. Significance levels: ***, *p* < 0.0005; ****, *p* < 0.00005. Total neurite length (**i**), territory area (**j**), and higher-order branch number (**k**) were all reduced in *CASK-*mutant neurons, whereas branch density (l) was increased. These data are from one of 9 independent experiments; see Additional file: Table A3 for analysis across all experiments

The abnormally small brains of *CASK*-mutant adults raise the question of when during development this size deficit arises. In mature larvae (w3L), freshly explanted CNS of *CASK* mutants (*∆18/∆18*) were visibly smaller than those of controls (*Ex33/Ex33*) of the same sex and developmental stage (Fig. 3a). Hence, microencephaly predates the onset of metamorphosis. Nonetheless, cellular densities after CNS dissociation and plating, as well as after culture for 3 div, did not differ between the two genotypes by holistic microscopic examination by an expert practitioner (J.A.T.; n = 9 independent experiments).

Unlike neuronal density, neurite arbors of *CASK*-mutant cultured neurons were obviously smaller after 3 div from those of controls. Qualitatively, the mutant arbors appeared “bushy”: relatively small and more compact (Fig. 3c-h). To quantify neurite-arbor morphogenesis in vitro, we compared primary cultures prepared from the entire CNS of *CASK*-mutant (*∆18/∆18*) and control (*Ex33/Ex33*) w3L using NeuronMetrics™ software to analyze digital images of immunofluorescently stained neurons sampled from each culture. (Fig. 3b; [115, 143]. For *CASK*-mutant arbors, total neurite length (Fig. 3i) and territory area (Fig. 3j) were reduced whereas branch density was elevated (Fig. 3l), all three with high degrees of statistical significance. Total branch and higher-order branch numbers (Fig. 3k) were reduced in most experiments. Primary process numbers were not significantly different between genotypes, suggesting that initiation of neurite outgrowth from *CASK*-mutant neuronal somata was not altered. The bushy phenotype is a population phenomenon, recognizable by microscopic inspection of individual dishes of cultured neurons. For single neurons, the differences from control are most apparent when comparing cells from approximately the same percentile of their distributions (e.g., Fig. 3c and f; d and g; e and h).

Because the entire CNS is dissociated, the cultures include diverse neuronal types with distinct in vivo morphologies (e.g., [67, 72, 136]. Histograms of neurite-arbor parameter distributions were unimodal for both *CASK* mutants and controls (Additional file: Fig. A2). This suggests that the bushy neurite-arbor defect is widespread rather than restricted to a small subset(s) of neurons, which would be consistent with the widespread *CASK* expression throughout the developing *Drosophila* CNS [32, 92, 96].

**Fig. 4:**
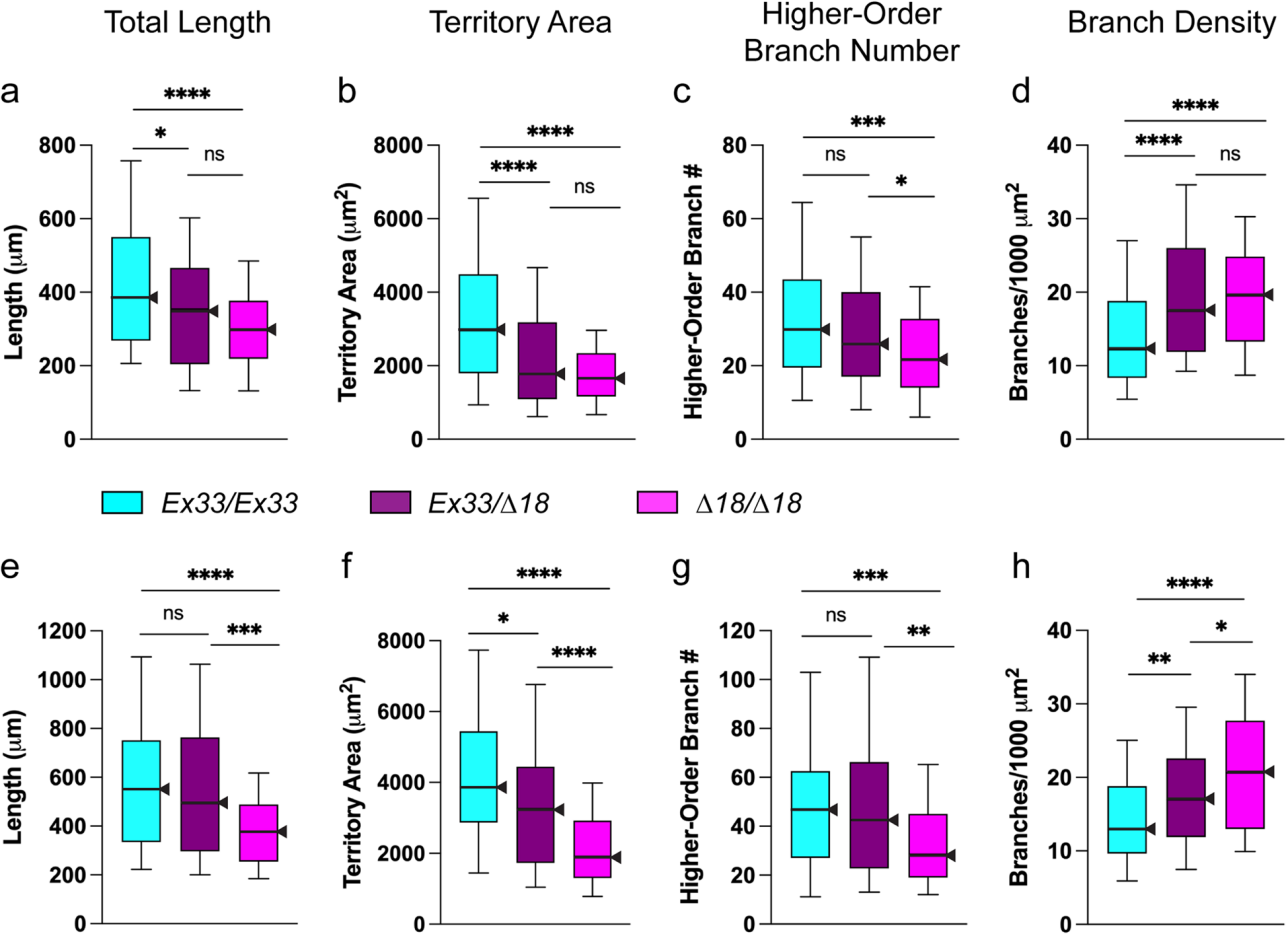
Analysis of *∆18* heterozygous neurons shows the bushy phenotype is neither strictly recessive nor dominant. Data from two independent experiments (**a**-**d** and **e**–**h**, respectively), each with parallel 3-div cultures of larval CNS neurons from *CASK* control (*Ex33/Ex33*; aqua), *CASK* mutant (*Δ18/∆18*; magenta), and heterozygote (*Δ18/Ex33*; burgundy). Box-plot distributions depicted as in Fig. 3, with *n* = 101–104 neurons from each genotype in each experiment. **a**, **e** Total neurite length; **b**, **f** territory area; **c**, **g** higher-order branch number; and **d**, **h** branch density. For two key characteristic features of the bushy phenotype, decreased territory area and increased branch density, *∆18* was dominant (**b**, **d**) or semi-dominant (**f**, **h**). Decreased total neurite length showed opposite results in the two experiments (**a**, **e**) and decreased higher-order branch number appeared to be recessive (**c**, **g**). Significance levels: *, *p* < 0.05; ***, *p* < 0.0005; ****, *p* < 0.00005

Quantitative data were obtained from nine pairwise comparisons (*∆18/∆18* vs. *Ex33/Ex33*) conducted over a period of several years with different laboratory locations, culture dishes, and biological reagents used (notably FBS). In addition, while most CNS dissociations were performed by manual trituration, some took place within microfluidic devices in a third location. As previously observed [76], there was considerable variation in the absolute values of morphometric parameters across experiments (e.g., Figs. 3i-l and 4a-h). For example, median total neurite length of control (*Ex33/Ex33*) arbors ranged from 305 µm to 652 µm. Nonetheless, the differences between genotypes were very consistent. For statistical analysis of data from all nine experiments, we used a linear mixed effects model in which each experimental median is considered a single sample (Additional file: Table A3). The differences between *CASK*-mutant and control neurite arbors were highly significant statistically, with magnitudes suggestive of biological significance: total neurite length (-35%; *p* = 0.0008), territory area (-48%; *p* = 0.0004), higher-order branch number (-30%; *p* = 0.0023) and branch density (+ 39%; *p* = 0.0006). Thus, the bushy phenotype of *CASK*-mutant neurite arbors represents a robust biological deviation from wild-type morphogenesis in vitro.

### The bushy phenotype is sensitive to *CASK* dosage

Human females with CASK-related disorders have semidominant mutations with strong evidence of haploinsufficiency and other indicators of dosage sensitivity (e.g., [57]. To explore potential genetic parallels between *CASK* in fly and human, we compared neurite arbors of three genotypes: homozygous control (*Ex33/Ex33*), homozygous hypomorphic mutant (*Δ18*/*Δ18*), and heterozygous (*Δ18/Ex33*). When a phenotype is strictly recessive, homozygous controls and heterozygotes consistently have the same (wild-type) characteristics; when a phenotype is strictly dominant, homozygous mutants and heterozygotes consistently have the same abnormal manifestations. In independent replicate experiments, we observed that *Δ18/Ex33* larval neurons grew arbors with median parameters between those of *Δ18*/*Δ18* and *Ex33/Ex33* homozygotes (Fig. 4a-h). In the statistical analyses, territory area (Fig. 4b, f) and branch density (Fig. 4d, h) values of *Δ18/Ex33* neurons were significantly different from both homozygotes in one experiment (Fig. 4f, h) but not significantly different from *Δ18*/*Δ18* mutants in the other experiment (Fig. 4b, d). Total neurite length of *Δ18/Ex33* was either not different from *Δ18*/*Δ18* mutant (Fig. 4a) or not different from *Ex33/Ex33* control (Fig. 4e). Thus, the features of the core triad of bushy neurite-arbors – decreased total length, decreased territory area, and increased branch density – do not behave like strictly recessive or dominant phenotypes. Rather these data suggest semidominance. In contrast, higher-order branch numbers, which across many experiments were less consistently reduced in *Δ18*/*Δ18* mutant neurite arbors, were not significantly different between *Ex33/Ex33* control and *Δ18/Ex33* neurons (Fig. 4c, g), suggesting this phenotype may be recessive.

**Fig. 5:**
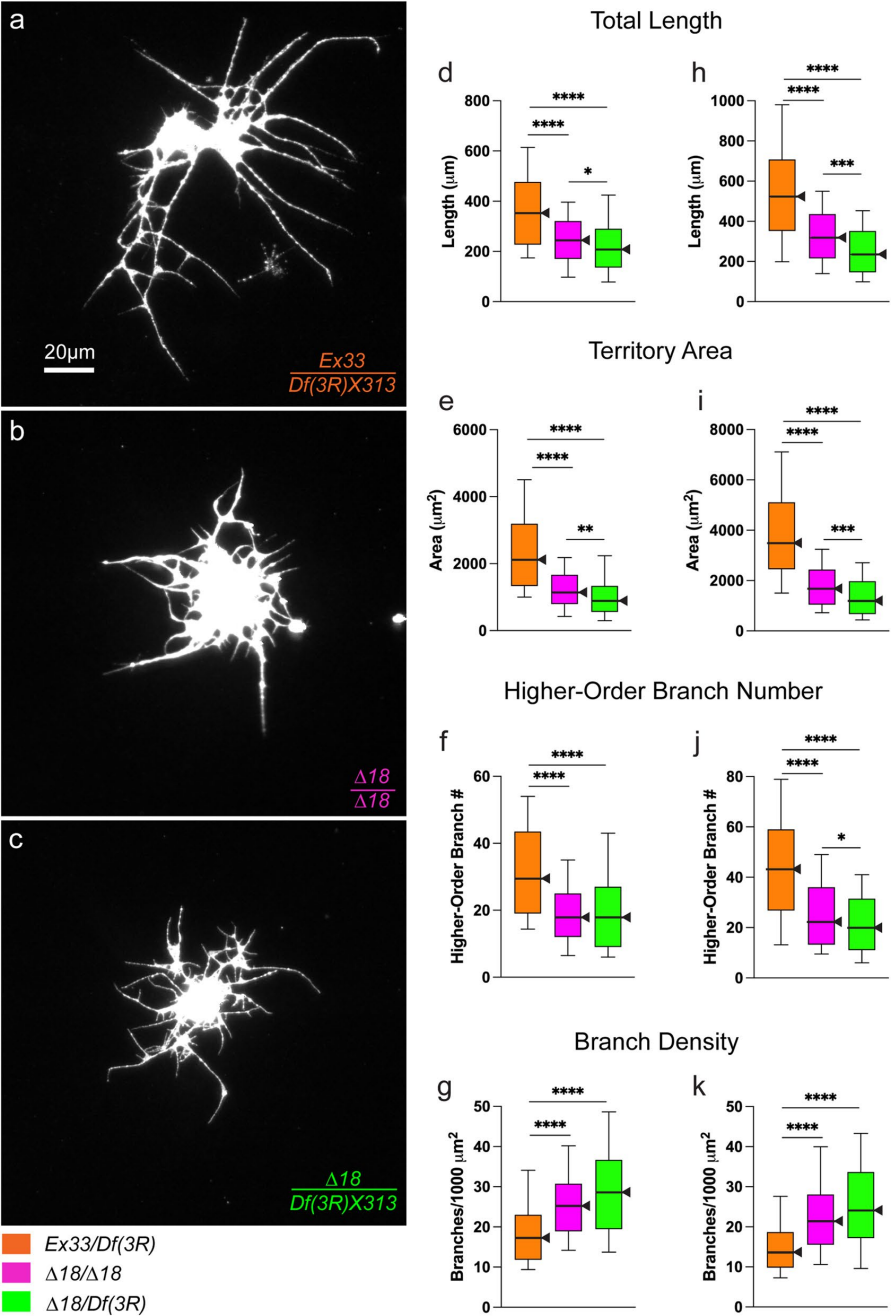
Chromosomal deficiencies of *CASK* reveal dosage sensitivity of the bushy phenotype. For two deficiencies, 3 div larval CNS cultured neurons were compared among three genotypes: *CASK* homozygous mutant (*Δ18/Δ18*), *CASK* hemizygous mutant (*∆18/Df*), and control allele over deficiency (*Ex33/Df*). **a**-**c** Photomicrographs of representative neurons, immunostained for neuronal membrane marker, from near the median for each of three parameters: total neurite length, territory area and branch density. 60X magnification; scale bar = 20 μm. **a** *Ex33/Df(3R)X313*. **b**-**c** Both *Δ18/Δ18* and *Δ18/ Df(3R)X313* neurons show bushy neurite arbors. **d**-**k** *CASK* hemizygous mutant neurons are more severely affected on most parameters. Quantification of neurite-arbor morphology; box-plot distributions depicted as in Fig. 3. Significance levels: *, *p* < 0.05; ***, *p* < 0.0005; ****, *p* < 0.00005. **d**-**g** *Ex33/Df(3R)X307* (*n* = 104; orange) compared with *Δ18/∆18* (*n* = 106; magenta) and *Δ18/ Df(3R)X307* (*n* = 106; green). **h**–**k** *Ex33/Df(3R)X313* (*n* = 106; orange), compared with *Δ18/∆18* (*n* = 105; magenta) and *Δ18/ Df(3R)X313* (*n* = 106; green)

To clarify the effects of *CASK*-gene dosage, we used two chromosomal deletions that remove all *CASK* function, *Df(3R)X307* and *Df(3R)X313*. With no *CASK*-null allele available, these are particularly valuable tools (Fig. 1b). Flies with compound heterozygous deletions [*Df(3R)X307/Df(3R)X313*] are viable as adults and sometimes called "*Caki* mutants" [96, 142]. Thus, unlike in humans and mice, *Drosophila CASK* is not essential for organismal viability. We used each deficiency chromosome to reduce *CASK* function beyond that in *∆18/∆18*, assessing neuronal phenotypes of *∆18* hemizygotes, *∆18/Df(3R)*. Because *∆18* is hypomorphic, we predicted the bushy phenotype would become more severe. For each deletion, we compared three genotypes (e.g., Fig. 5a-c):

1. hemizygous control: *w/* + or* w/w;* + *; Ex33/Df(3R)*
2. homozygous mutant:* w/w;* + *; ∆18/∆18*
3. hemizygous mutant:* w/w;* + *; ∆18/Df(3R)*

For both deficiencies, arbors of hemizygous mutant neurons showed highly significantly reduced total neurite length, territory area, and higher-order branch number, along with increased branch density compared with hemizygous controls (Fig. 5d-k)*.* Moreover, hemizygous mutant neurons were at least as severely affected as homozygous *∆18* mutant neurons. For both deficiencies, this was most evident for total neurite length and territory area, which were significantly smaller in *∆18/Df(3R)* than in *∆18/∆18* (Fig. 5d-e, h-i). Hence, the bushy phenotype shows a severity range inversely related to *CASK*-gene dosage (in decreasing order of phenotypic severity): *∆18/Df(3R)* > *∆18/∆18* > *Ex33/∆18* > *Ex33/Ex33*.

### Transgenic *CASK*^+^ improves the bushy phenotype

A definitive method for proving a phenotype is due to LOF of a specific gene is to demonstrate rescue by a wild-type transgene. We therefore tested whether wild-type *CASK* would rescue neurite-arbor size and shape in *∆18* homozygotes. The degree of rescue may depend on the nature of the mutation and the genetic reagents used. We used *elav-GAL4*^*C155*^ to drive expression in neurons of a single-copy transgene, *UAS-CASK*^+^, a cDNA encoding full-length wild-type CASK. For two reasons, we expected that rescue might be strong but incomplete. First, the phenotype is dosage-sensitive (Figs. 4 and 5). Second, *elav-GAL4*^*C155*^ drives expression in a large majority of cultured neurons (~ 85%; see Methods), but is not fully pan-neuronal in vitro. Hence, we predicted that one transgenic copy of *CASK*^+^ would provide partial restoration of *CASK* function and yield an intermediate phenotype, better than that of *∆18* homozygotes, but not as good as control (*Ex33*) homozygotes.

We first tested whether either of the two transgenes would impact the bushy phenotype of *CASK*-mutant *∆18* homozygotes. A three-way comparison was performed as follows:

1. homozygous mutant: *w/w;* + *; ∆18/∆18*
2. driver transgene in mutant background: *elav-Gal4*^*C155*^* w/w;* + *; ∆18/∆18*
3. target transgene in mutant background: *w/* + *; UAS-CASK*^+^*/* + *; ∆18/∆18*

**Fig. 6:**
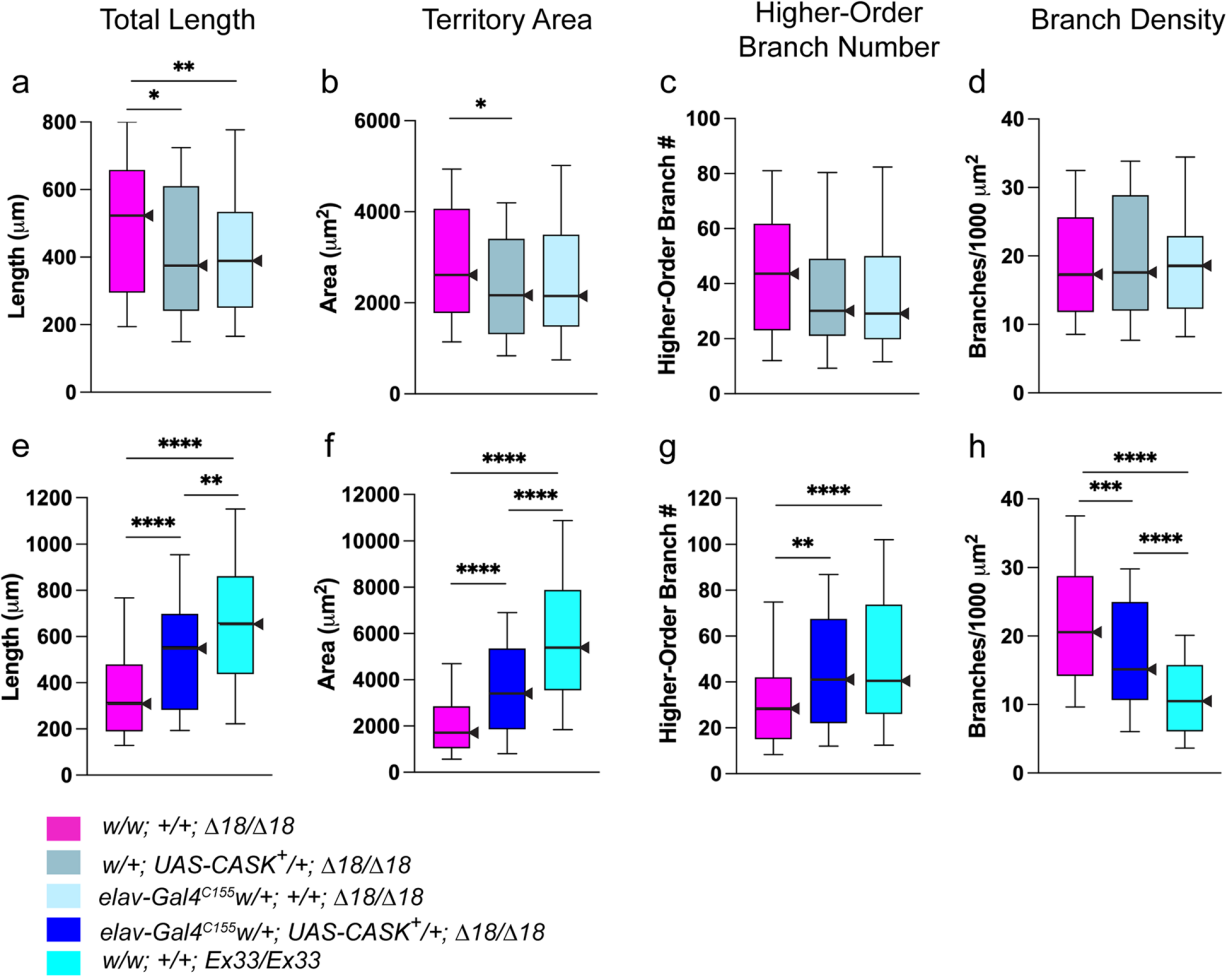
Transgenic expression of *CASK*^+^ significantly improves the bushy phenotype. Neurite-arbor morphology parameters from 3 div larval CNS neuronal cultures. Box-plot distributions depicted as in Fig. 3. Significance levels: *, *p* < 0.05; **, *p* < 0.005; ***, *p* < 0.0005; ****, *p* < 0.00005. **a**-**d** Neither individual transgene, UAS-controlled wild-type full-length *CASK* cDNA or the neuronal driver, *elav-Gal4*^*C155*^, improved the bushy phenotype of *CASK*-mutant (*Δ18/Δ18*) neurons. Three genotypes were compared: *w/w;* + *; Δ18/Δ18* (*n* = 104; magenta); *w/* + *; UAS-CASK*^+^*/* + *; ∆18/∆18* (*n* = 104; gray); and *elav-Gal4*^*C155*^* w/* + *;* + *; ∆18/∆18* (*n* = 105; light blue). **a** Both transgenes caused modest reductions in total neurite length, i.e., worsening the phenotype. **b** Territory area was mildly reduced by *UAS-CASK*^+^ only. **c** Higher-order branch number was not affected by either transgene. **d** Branch density, the most distinctive feature of the bushy phenotype, was not affected by either transgene. **e**–**h** Driving expression of transgenic *CASK*^+^ in neurons significantly improved all four parameters of the bushy phenotype. Three genotypes were compared: *w/w;* + *; Δ18/Δ18* (n = 105; magenta); *elav-Gal4*^*C155*^*, w/* + *; UAS-CASK*^+^*/* + *; Δ18/Δ18* (n = 104; bright blue); and *w/w;* + *; Ex33/Ex33* (*CASK* control; *n* = 105; aqua). **e** Total neurite length. **f** Territory area. **g** Higher-order branch number. **h** Branch density. Strong but incomplete transgenic rescue is consistent with the dosage-sensitive nature of the bushy phenotype

Neither of the two transgenes improved the neurite-arbor phenotype of *CASK ∆18* homozygous mutants (Fig. 6a-d). In fact, both transgenes caused modest worsening, i.e., reductions in neurite-arbor size beyond that of homozygous mutant neurons. Total length was significantly reduced by both *UAS-CASK*^+^ and *elav-GAL4*^*C155*^; territory area by *UAS-CASK*^+^ only. Either or both of these effects could make detection of phenotypic rescue more difficult, at least for arbor size. Neither transgene affected branch density (one of the most characteristic features of the bushy phenotype) or higher-order branch number.

To test for transgenic rescue of the bushy phenotype, three replicate experiments, performed in parallel to minimize biological and technical variables, compared neuronal cultures from three genotypes:

1. homozygous control:* w/w;* + *; Ex33/Ex33*
2. homozygous mutant:* w/w;* + *; ∆18/∆18*
3. experimental: *elav-Gal4*^*C155*^* w/* + *; UAS-CASK*^+^*/* + *; ∆18/∆18*

Expression of a single wild-type *CASK*^+^ cDNA transgene in the large majority of *∆18* homozygous mutant neurons resulted in highly significant increases in total neurite length, territory area, and higher-order branch number, along with decreases in branch density (Fig. 6e-h). In other words, the bushy phenotype was significantly improved, with neurite-arbor parameters intermediate between those of mutant and control homozygotes. This highly significant partial rescue is strong confirmatory evidence that the bushy phenotype maps to *CASK* and results from *CASK* LOF.

### Optimization of microfluidic technology for CNS tissue dissociation

The robust nature of the bushy phenotype, with visible and quantitative features, makes it a strong candidate for phenotype-based screening for drug discovery. We previously demonstrated that primary neuronal cultures can be screened for compounds that modify a neurite-arbor defect [75]. However, manual dissociation is a major obstacle because it is highly fatigable, non-trivial to learn, and uncontrolled in terms of shear stress applied to the tissue. In fact, the shear stress generated during manual trituration has never been estimated. The rate-limiting step of CNS tissue dissociation must be overcome before screening speed can be scaled up toward high throughput. Microfluidic technology has the potential to solve this problem. Manually dissociated cells from prenatal spinal cord were sorted within a microfluidic device prior to culture [162]. Several groups reported dissociation of non-neural-tissue into viable cells – rodent kidney, liver, heart, adipose tissue, and tumors, as well as human placenta and endometrium [3, 5, 93]. Relative to other cell types, the unique structure of neurons adds a challenge – axons and dendrites are severed during dissociation and the neuronal membrane must reseal at each detachment point.

We previously reported initial success with development of a mechanized, observable, standardized – and therefore non-fatigable – microfluidics-based method for dissociation of *Drosophila* larval CNS into viable neurons for primary culture [68]. Enzyme-treated tissue was exposed to a controlled oscillating fluid flow that generates a periodic stress field in a microchannel, yielding single-cell suspensions. Here we report on optimization of device design and operation for dissociating *Drosophila* larval CNS tissue. We also demonstrated utility for dissociation of developing rodent CNS.

**Fig. 7:**
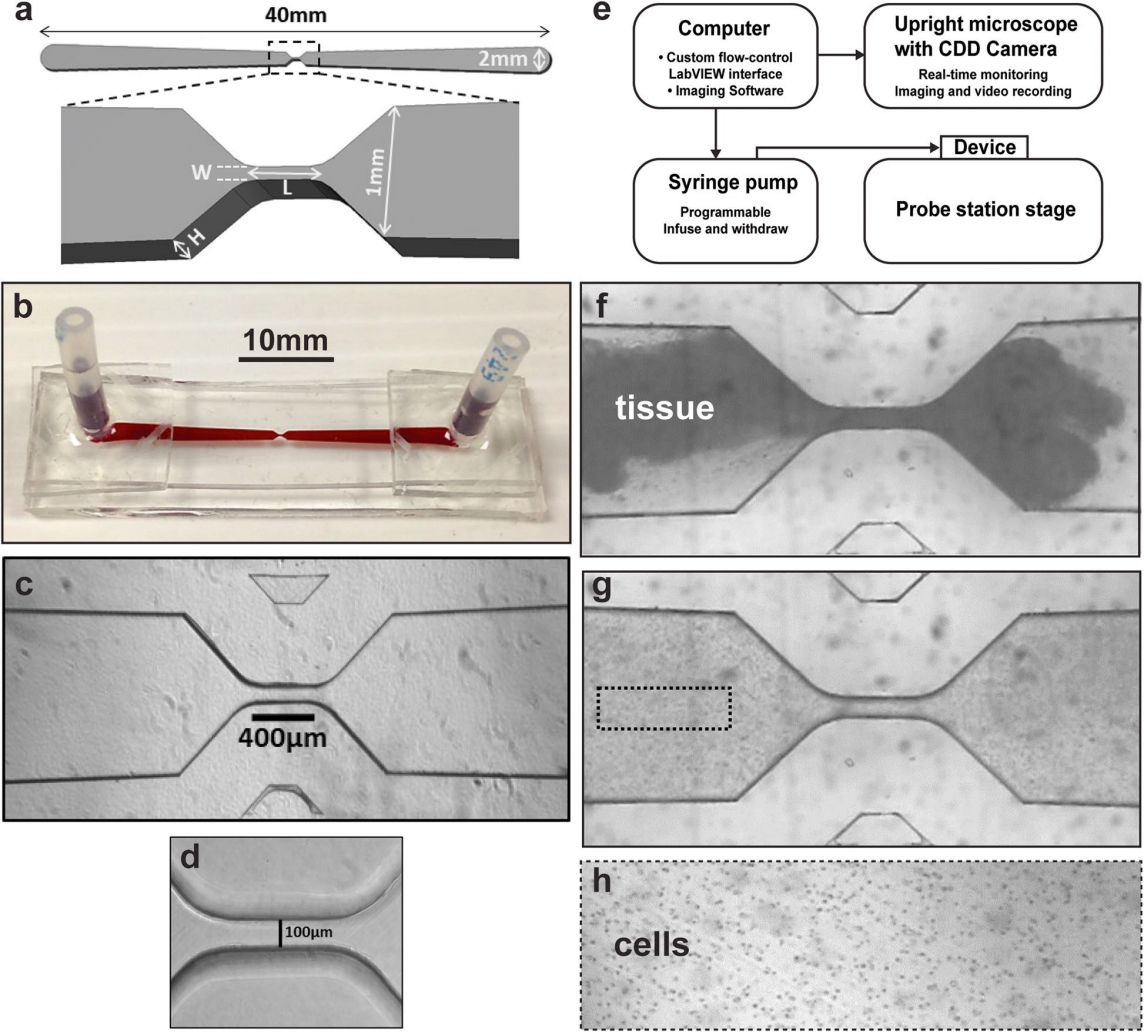
A microfluidic system for dissociation of CNS into viable neurons. **a** Schematic drawing of the microdevice, top-down view, with central portion magnified and shown in oblique lateral view. Tissue dissociation takes place in the narrow central orifice. Length (L), width (W), and height (H) were varied during optimization. **b** Photograph of single-channel microfluidic device, lateral oblique view: ports extending upward from the distal ends provide inlet/outlet access and connection to the pump. The channel has been filled with red dye to make it easily visible. **c**, **d** Photomicrographs of the central part of the channel, top-down views. **c** As the main channel segments approach the central orifice, the walls narrow at 45° angles. **d** High-magnification view of the orifice. Note the smoothness of the channel walls. The 100-µm-wide channels were not needed or used in the experiments reported here. **e** A block diagram illustrating the main components of the experimental system. The microfluidic device sits on the stage of a Signatone probe station equipped with an upright compound microscope and computer-controlled CCD camera. One device port is connected by tubing to a computer-controlled syringe pump that drives oscillatory flow within the channel. **f**–**h** Individual frames of video acquired during the dissociation of an enzyme-treated piece of rat E18 hippocampus in a microfluidic device. **f** Intact tissue is driven by the pump through the central orifice with dimensions L = 400 µm, W = 70 µm. **g** Following oscillatory cycles of flow-induced shear stress (flow rate 50 µl/sec, infusion volume 12.5 µl, 4 Hz), the tissue has been dissociated into cell clusters and single cells. Dotted rectangle indicates approximate size of the area shown in (h) at a later time at higher magnification. **h** Additional cycles have completed the dissociation into single cells which were collected and plated for culture

The microfluidic device conceptual design consists of channels with mirror-image-symmetric layout centered on a narrow constriction zone (“orifice”) (Fig. 7a, b). Transparency of the device allows for real-time monitoring by microscopy. One port is connected to a computer-controlled pump to manipulate the flow field while the other port is used for tissue loading and cell recovery (Fig. 7b, c). Tissue dissociation, driven by bi-directional oscillatory fluid flow, occurs in the narrow central orifice (Fig. 7d) within which shear-stress magnitude and gradients are enhanced.

Devices fabricated with varying channel height and central-orifice length and width (Fig. 7a), and operated under varying flow parameters to optimize dissociation of fruit fly CNS tissue. Once we could achieve apparently full dissociation, by microscopic examination in real time within the device, the outcome measures were (1) completeness of dissociation to single cells, which could be assessed after plating the cells and flooding the dishes, and (2) neuronal health, as evidenced by survival and neurite arbor morphogenesis during culture for several days. For most of the optimization trials, outcome assessments were conducted by daily holistic scoring by an expert in manual dissociation (J.A.T.). Once device and flow parameters were identified that yielded excellent Drosophila CNS dissociation, cell recovery, and neurite outgrowth, as assessed by holistic scoring, device trials were performed in parallel with manual dissociation, with the cultured neurons immunostained and imaged for quantitative comparison.

While the orifice length was fixed at 30 µm in previously reported work [68], it was increased to 50, 100, 200, or 400 µm in the current trials, with the longest yielding better and more consistent results. Hence, the large majority of experiments were conducted with an orifice length of 400 µm. Note that the maximum dimension of the *Drosophila* larval CNS is ~ 500 µm. The widths of the central orifice tested were 25, 40, 50, 60, 70, and 80 µm. The channel/orifice heights tested were 350, 400, 450, 490, and 500 µm. The resulting peak shear stress across all trials and tissues ranged from 10 to 10^4^ dynes/cm^2^. The infusion volumes tested were 10.0, 10.3, 10.4, 10.5 and 12.6 µl, at oscillation frequencies of 3–5 Hz. The flow rates were 50–60 µl/s, occasionally increased to 80 µl/s during the dissociation. The number of cycles varied from ~ 300 to ~ 2,000, with > 15 points along the range tested.

For *Drosophila* larval CNS, optimal channel height was 450 µm; optimal orifice dimensions were 400 µm in length and 50 µm in width. Optimal flow parameters were determined to be 10.5 µl for the infusion volume, at 50 µl/sec flow rate, with an oscillation frequency of 5 Hz for 1,400 cycles; total time was ~ 5 min. The resulting dissociations were highly consistent. Within the first 1–2 min, the tissue was reduced to a suspension of cell clusters and individual cells. By the end, the dissociations were complete with few, if any, residual clumps or clusters of cells. Under these optimal conditions, the tissue was transiently exposed to an estimated maximum shear stress of ~ 2000 dynes/cm^2^ based on the model presented in Jiang et al. [68]. Compared with our standard manual dissociation protocol [76, 143], the device-based oscillation rate is about five times faster and the cycle number is ~ 17 times higher. These parameters would be impossible to achieve with a manual pipetting device.

Several hours after plating the device-dissociated *Drosophila* larval CNS cells, immunostaining for standard markers revealed that the large majority were neurons, with a small number of glia and some presumed neural stem cells (data not shown). Neuronal health, as evidenced by neurite-arbor morphogenesis during the next several days, was excellent (see next section). As in the case of manually dissociated CNS [143], glia were not present at 3 div. Hence, these are close-to-pure neuronal cultures, with the neurons having a wide range of in vivo developmental histories.

Following optimization of device-based dissociation of fruit fly larval CNS, we tested prenatal (E18) rodent hippocampal tissue. Individual tissue pieces were loaded into each channel (Fig. 7d). Those were at least several fold larger than the maximum dimension of a *Drosophila* larval CNS. Therefore, the channel height was increased to 500 µm, with orifice width increased to 70 µm and orifice length maintained at 400 µm. Similarly, compared to the Drosophila protocol, the infusion volume was increased to 12.5 µl (and sometimes as high as 30 µl), with oscillation frequency somewhat slower at 3–4 Hz for ~ 1,000 cycles. Flow rates of 50–100 µl/sec were effective; dissociation times were 3–6 min. Following dissociation, the estimated cell yield from a pair of E18 rat hippocampi was 1.8 million, which compares favorably with BrainBits®/Transnetyx® (https://tissue.transnetyx.com/E18-Rat-Hippocampus_4, last accessed 6 December 2022) guarantee of 1 million cells following manual dissociation. After plating, the dissociated hippocampal neurons showed qualitatively good survival and extension of complex arbors (Additional file: Fig. A3). While we did not undertake a full optimization, we did demonstrate that microfluidic dissociation has utility for mammalian CNS.

### A microfluidic system for CNS-tissue dissociation reproduces the bushy phenotype of *CASK*-mutant neurons with high fidelity

**Fig. 8:**
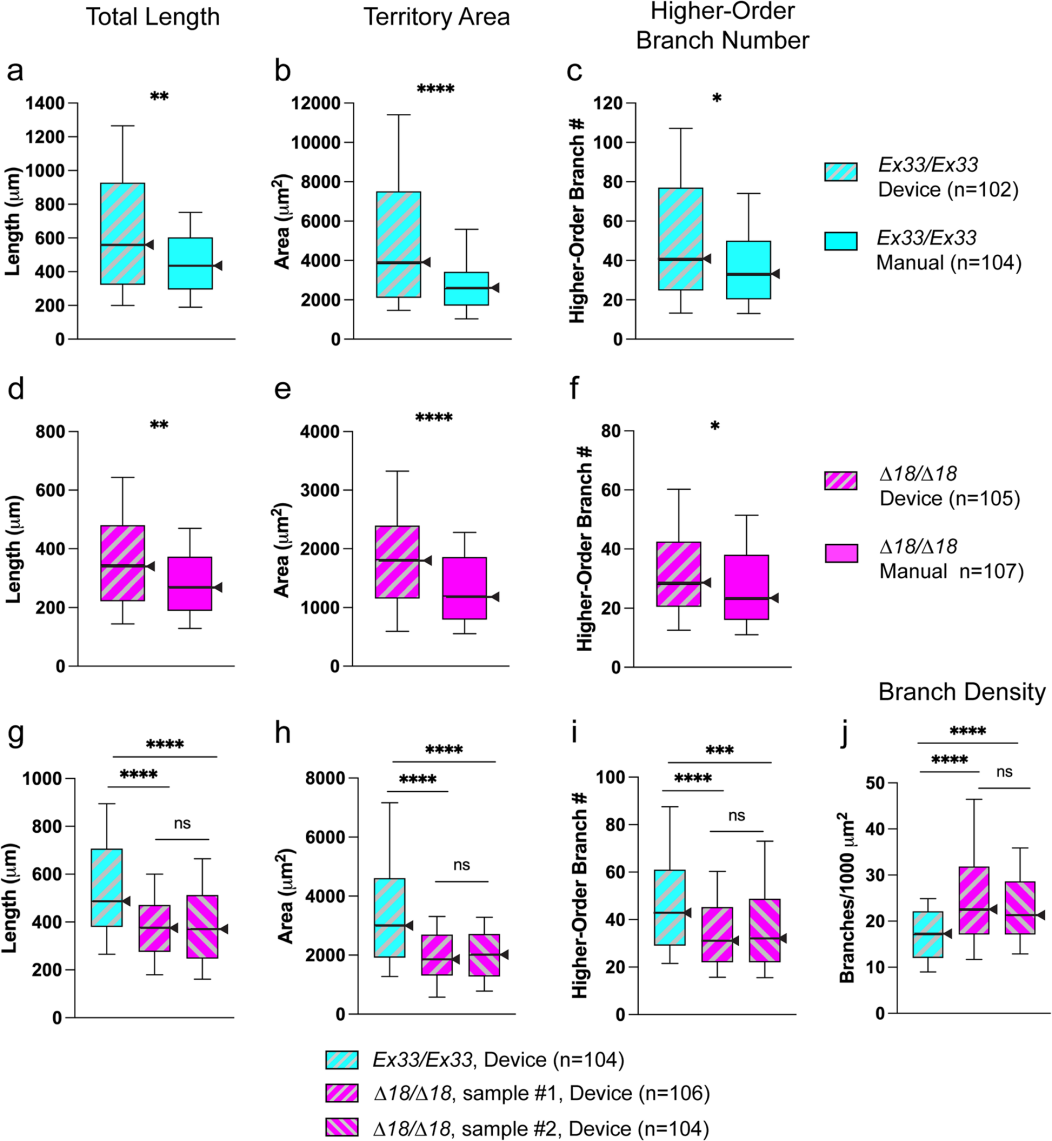
Device-dissociated neurons extend larger arbors and manifest the *CASK*-LOF bushy phenotype in vitro. Device dissociations were performed with flow parameters 50 µl/sec, infusion volume 10.5 µl, 4.8 Hz, mean cycle number1444. Neurite-arbor size parameters from 3 div cultures, comparing methods of dissociation and genotypes. Box-plot distributions depicted as in Fig. 3. Significance levels: *, *p* < 0.05; **, *p* < 0.005; ***, *p* < 0.0005; ****, *p* < 0.00005; ns, not significant. **a**-**f** Device vs. manual dissociation. **a**-**c** *Ex33/Ex33*. Device dimensions: channel height 500 µm; orifice length 400 µm; orifice width 60 µm. **d**-**f** *∆18/∆18*. Device dimensions: channel height 450 µm; orifice length 400 µm; orifice width 50 µm. For both genotypes, total neurite length (**a**, **d**), territory area (**b**, **e**), and higher-order branch number (**c**-**f**) were significantly higher for microfluidic device-dissociated neurons. **g**-**j** Comparison of device-dissociated neurons from control (*Ex33/Ex33*; one sample) and *CASK* mutant (*∆18/∆18*; two samples) larval CNS. Device dimensions: channel height 450 µm; orifice length 400 µm; orifice width 50 µl. **g** Total neurite length. **h** Territory area. **i **Higher-order branches. **j** Branch density. Device-dissociated *CASK*-mutant neurons extend bushy neurite arbors, replicating the phenotype seen in manual cultures. In addition, two cultures prepared after serial device-based dissociation show marked consistency of neurite-arbor parameters

When pairs of *Drosophila* neuronal cultures were prepared from CNS of the same genotype, dissociated in parallel in a microdevice versus manually by an expert (J.A.T.), the device-dissociated neurons grew significantly larger neurite arbors (Fig. 8a-f). This was particularly striking for territory area, but total neurite length and higher-order branch number were also increased. The neurite-arbor size difference was evident in neurons of both *CASK* genotypes (control Fig. 8a-c; mutant Fig. 8d-f). These findings suggest that the device-dissociated neurons were healthier, perhaps because they experienced less mechanical trauma. Comparing cultures of neurons dissociated in a single-channel device with those dissociated immediately thereafter in a device consisting of two identical, parallel dissociation channels, we found that neurite-arbor parameters did not differ between the two (Additional file: Fig. A4).

In order to make use of the benefits of microdevice-based CNS dissociation, especially for drug screening and studies of *CASK* function, it is essential to determine whether the method modifies the *CASK*-LOF mutant neuronal phenotype. This was of particular concern because of the relatively large arbor size of device-dissociated cultured neurons (Fig. 8a-f). Dissociations were performed in series in single-channel devices using larval CNS tissue from two different *CASK* mutants (*∆18/∆18*) and one control (*Ex33/Ex33*). Compared with control neurons, the *CASK*-mutant neurons extended smaller, denser neurite arbors, with high levels of statistical significance for all parameters (Fig. 8g-j). This replicates the *CASK*-mutant bushy phenotype. In *CASK*-mutant cultures from the two sequential dissociations shown, device-dissociated *∆18/∆18* neurons had markedly similar neurite arbors with no statistically significant differences in any parameter. Hence, device dissociation yields excellent consistency and does not alter the *CASK* phenotype.

## Discussion

### The challenge of reconciling CASK biochemistry, cell biology, and human genetics

CASK defies simple classification. It is a highly conserved member of the MAGUK (Membrane-Associated Guanylate Kinase) protein family [31, 82], this study), based on the presence and order of its three C' domains: PDZ, SH3 (Src homology 3) and GUK (guanylate kinase). Like other MAGUKs [44], CASK acts as a scaffold that tethers and localizes adhesion molecules, receptors, and signaling molecules [59, 106]. However, the name CASK, an acronym for calcium/ calmodulin-dependent serine protein kinase, is based on sequence similarity of its N'-terminal domain, which is unique among MAGUK family members [53]. Given that CASK does not have typical CaMK activity, this name is problematic. CASK was classified as a pseudokinase because its CaMK-like N' domain lacks a DFG motif (Asp-Phe-Gly) which is required for Mg^2+−^binding and was thought to be essential for kinase activity [12]. However, CASK's CaMK-like N' domain can catalyze Mg^2+^-independent phosphorylation [109] of neurexin-1, a CASK binding partner [53]. Thus, CASK has at least two distinct molecular functions, scaffolding and enzymatic, both involving presynaptic neurexin. The third is transcriptional regulation, via partnership with co-activators [59].

The human phenotypes of *CASK*-related disorders have a remarkable range of clinical severity and manifestations, none of which has been conclusively ascribed to specific molecular functions. The most consistent phenotypes are intellectual disability (ID) and microcephaly with hindbrain predominance. Microcephaly is common in monogenic ID disorders (occurring in ~ 40% based on OMIM Clinical Synopsis pages; data not shown), as is variable expressivity and incomplete penetrance. Indeed, most of the disorders caused by mutations in CASK partner genes have microcephaly as a feature (Additional file: Table A1). Among children with *CASK*-related disorders, progressive postnatal microcephaly has been extensively documented. In addition, congenital microcephaly has been reported in a substantial minority of cases (e.g., [15, 104, 112, 134, 149]. Hence, CASK function in the human CNS starts prenatally, aligning with highest *CASK* transcript accumulation in the prenatal brain and a gradual decline in all brain regions over the lifespan (Human Brain Transcriptome project, https://hbatlas.org/hbtd/images/wholeBrain/CASK.pdf, last accessed 3 January 2023; [69]. *CASK* is expressed in many other organs as well (NCBI; https://www.ncbi.nlm.nih.gov/gene/8573, last accessed 10 January 2023). Common phenotypes outside the CNS may include short stature and mild facial dysmorphism [103, 104, 149].

Much of the molecular and cellular analysis of CASK function has focused on neurons at advanced stages of differentiation to study synapse formation and physiology, sometimes alongside investigations of partner proteins [59]. This focus reflects the history of CASK's discovery, its localization to both sides of the mature synapse, and its binding to numerous synaptic proteins. For example, based on acute RNA interference, cultured hippocampal neurons required CASK for normal size and density of dendritic spines [19]. Cultured cortical neurons from transgenic *CASK*-KO newborn mice showed abnormal rates of spontaneous synaptic release events, elevated for excitatory (glutamatergic) and reduced for inhibitory (GABAergic), but with no change in evoked release or synapse ultrastructure [8]. Similarly, in acutely cultured brain slices from heterozygous *CASK*-KO mice (functionally mosaic, like human females with MICPCH), miniature excitatory/inhibitory (E/I) postsynaptic currents were increased/decreased, respectively, but only in the CASK-deficient neurons [105]. Retinogeniculate synapses of *CASK*-heterozygous mice showed reductions in active zone numbers [88]. Synaptic physiology abnormalities were also demonstrated in *CASK*-LOF *Drosophila* [21, 48, 147, 168]. When human embryonic stem cells (hESC) were used to create a *CASK*-null condition by gene editing, the mutant cortical excitatory neurons differentiated from them showed defective synaptic transmission and network activity [99]. Evidence of E/I imbalance in humans with *CASK*-related disorders came from synapses of neurons differentiated from *CASK*-mutant induced pluripotent stem cells (iPSc), showing reduced size of inhibitory presynaptic sites, as well as from in vivo brain imaging by magnetic resonance spectroscopy showing low levels of GABA [10].

On the other hand, synaptic dysfunction cannot explain reduced brain size especially when it starts in utero. The brainstem and cerebellum are exquisitely sensitive to *CASK*-LOF, with markedly reduced volume detectable in early postnatal MRIs of children with MICPCH. The brainstem defects are believed to cause hypoventilation, potentially fatal [8]. Two neonatal autopsies revealed minimal formation of cerebellar folia, marked loss of cerebellar granule cells, and astrogliosis consistent with prior neurodegeneration [112, 120]. The hypoplasia of severe MICPCH extends beyond hindbrain structures. Published MRIs show cortical volume reduction, simplification of gyral patterns, and corpus callosum abnormalities (e.g., [57, 103, 108, 112, 113, 118, 128, 148]). The variable phenotypes of optic nerve hypoplasia and retinopathy [71, 80, 112] also reflect disrupted early forebrain development. By histopathology of a two-week-old male, the cerebral cortex showed lamination defects selectively affecting layers V and VI [112]. These layers express TBR1, a transcriptional co-activator which partners with CASK to up-regulate *RELN* expression [60]. These phenotypes are not causally related to proposed synaptic functions of CASK. Indeed, analysis of several types of transgenic *CASK*-mutant mice implicate non-neuronal cells as contributors to microcephaly [145].

As for neuronal microanatomy, there are no reported Golgi-impregnation studies in any species with *CASK* LOF. McSweeney et al. [99] evaluated neurite arbors of *CASK-KO* neurons differentiated from gene-edited hESC. Early during differentiation, neurons showed increased dendritic branch points; dendritic length was not consistently altered and branch density was not reported. In co-cultures with murine wild-type glia, mature neurons had normal-sized dendritic arbors. Increased branch complexity of differentiating *CASK-KO* human neurons in vitro aligns with the bushy phenotype.

The CNS phenotypes of *CASK*-related disorders are likely to result from both cell-autonomous and non-cell-autonomous primary defects, with a cascade of secondary effects on brain development, worsened by functional disruptions of neural circuitry [107]. For example, *CASK*-LOF altered transcript profiles in mouse brain [121], in neurons differentiated from patient-specific iPSc lines or siRNA-mediated knockdown in controls [10] or differentiated from gene-edited hESC [99]. In *CASK*-heterozygous mouse brain, where X inactivation leads to interactions between wild-type and mutant cells, marked changes in the proteome, affecting many pathways, suggest a stepwise expansion of the effects of *CASK-*LOF [121]. Non-cell-autonomous effects could arise by cell–cell interactions, e.g., trans-synaptic or neuron-glia. Impaired metabolism has also been reported in *CASK*-heterozygous mice [145]. In contrast, Drosophila *CASK* mutants have normal oxygen consumption on a per-mg basis [42].

### Multi-level alignment of human and fruit fly CASK: genetics and neurobiology

The value of the *Drosophila* genetic model system for studying neurodevelopmental disorders is well-recognized [7, 22, 90, 97]. Work from multiple groups has documented cognitive and motor-behavior phenotypes in *Drosophila* with *CASK*-LOF or absence [6, 48, 95, 96, 142]. When the design of those studies allowed, behavioral phenotypes were sensitive to *CASK* dosage, as in humans. Memory or learning defects in *CASK*-LOF larvae or flies using appetitive or aversive associative learning paradigms [48, 95] provide face validity for the ID of *CASK-*related disorders. Stereotyped repetitive grooming behavior may be relevant to a core symptom of ASD [6], which can be seen with human *CASK*-LOF [10, 64, 139].

At the biochemical level, *Drosophila* CASK promotes autophosphorylation of CaMKII. In *Caki* mutant flies (with overlapping deletions), human *CASK*^+^ can rescue the CaMKII-autophosphorylation defect [48]. Like mammalian CASK, the fruit fly ortholog has many binding partners,a majority are specific to neuronal subsets [110]. X inactivation is not an issue, because *Drosophila CASK* is not X-linked and the mechanism of dosage compensation is different. The lack of mosaicism in *CASK* heterozygous *Drosophila*, cf., in human females, could be of benefit.

We have discovered a small-brain phenotype in *Drosophila* with *CASK*-LOF. This is evident by eye midway through development (Fig. 3a) and persists through late metamorphosis (Fig. 2e). Thus, the requirement of *CASK*^+^ for normal brain-size acquisition is highly conserved among humans [102], mice [145], zebrafish [24], and now fruit flies. We found no obvious indication of reduced neuronal numbers in *CASK*-mutant larval CNS. Blinded full-dish counts of cultured neurons of three genotypes showed only modest differences (within 4–5%) among *∆18/∆18* mutant, *Ex33/Ex33* controls, and *∆18/∆18* with one copy of *CASK*^+^ (data not shown). With the caveat that neurogenesis and programmed cell death have not been assessed, these preliminary data suggested that diminished neuron numbers do not explain the gross reduction in *CASK*-mutant CNS size.

We also demonstrated that acquisition of normal *Drosophila* body length requires *CASK*^+^ function (Fig. 2c). This is consistent with a statistically significant reduction in body weight of *∆18/∆18* compared with *Ex33/Ex33* [42]. The similarity of puparium length and mammalian CRL as measures of axial growth has several dimensions. In humans, first-trimester CRL reflects gestational age [123]. Conversely, reduced CRL may be associated with developmental abnormalities of genetic or environmental origin [11, 54, 138, 164]. A set of phylogenetically conserved transcription factors, originally discovered in *D. melanogaster*, control growth and development along the longitudinal axis of insects and vertebrates [18, 50]. Thus, while not precisely analogous to stature, puparium length nonetheless provides an excellent measure of fruit fly body length at a midway point during development. The combination of microcephaly, a form of 'short stature', and impaired cognition represents strong face validity of the *Drosophila* model of *CASK*-related disorders. To understand the cellular basis of microencephaly in *CASK*-LOF *Drosophila*, we turned to primary neuronal cell culture.

### The bushy phenotype of *CASK*-mutant neurite arbors

"Bushy" is a specific small-arbor phenotype in which primary size parameters are reduced but with increased branch density (e.g., Fig. 3i-l), including both branches per unit area and branches per unit length (Additional file: Table A2). Qualitatively, the bushy phenotype is reminiscent of the *tumbleweed*-mutant dendritic arbors of sensory neurons [46]. Three other small-arbor mutant phenotypes of that we have characterized in vitro have markedly different features. *Pak* LOF causes small, sparse arbors [87], while *CBP/nejire* LOF causes a very severe 'naked-arbor' defect (K. Olsen, R. Kraft, and L.L.R., unpublished). Finally, *fascin/singed* LOF causes the "filagree" phenotype, with marked curvature of neurites and a small territory despite normal length [74, 75].

Because primary neurite numbers were unchanged by *Drosophila CASK* LOF, while higher-order branches and total neurite length were markedly reduced, we infer that neurite initiation from the neuronal cell body was normal, but neurite elongation was deficient. We further infer that branch formation, either interstitial or at growth cones, occurred prematurely, leading to increased branch density. Because our cell culture system is low density and nearly-all-neuronal, bushy neurite arbors must reflect intrinsic properties of mutant neurons. However, they may not be strictly speaking cell-autonomous because prior to dissociation, the neurons had spent considerable developmental time in contact with each other and with glia. Hence, the bushy phenotype may derive in part from cell–cell interactions earlier in life.

We hypothesize that the in vitro bushy phenotype reflects small dendritic and axonal arbors in vivo which, in turn, contribute to microencephaly in *CASK*-LOF *Drosophila*. In preliminary studies staining for the presynaptic marker Bruchpilot, we noted that larval CNS of *∆18* homozygotes appeared to have reduced synaptic neuropil volume relative to brain-lobe volume (data not shown). It may also be that the impact of modestly reduced neuronal number is amplified by reduced arborization within neuropils.

To our knowledge we are the first to report on neurite-arbor morphogenesis in primary neuronal cultures prepared from animals with germline *CASK*-LOF mutations. The bushy phenotype has two mammalian correlates. First, in a *CASK*-overexpression context, transfection with wild-type *CASK* promoted neurite outgrowth by cultured neurons differentiated from mouse Neuro2A cells [157]. Second, short-term culture of neurons differentiated from gene-edited *CASK*-*KO* hESC showed increased neurite branching [99]. Using a different protocol that included wild-type mouse glia, longer-term neuronal cultures had normal branching (*op. cit.*), perhaps due to rescue by glia.

Elaboration and pruning of cortical neuron dendritic arbors are phases of brain development that, of necessity, impact the architecture of neural circuits. These differentiation processes are disrupted in many ID disorders, resulting in abnormalities of dendritic arbor size and shape [30, 70, 78, 125]. In conjunction with abnormal axonal projections, notably those involving the corpus callosum [35], faulty dendritic arbors plausibly contribute to functionally altered neural circuits and underlie deficits in cognitive development [125]. In *Drosophila Pak*-mutant neurons, the sparse neurite arbors seen in vitro [87] align with the reduced cortical neuron dendritic arbors of mouse *Pak* mutants in vivo, likely via actin cytoskeleton dysregulation [63]. Males with *PAK3* mutations have ID with microcephaly (MIM #300558; https://omim.org/entry/300558, last accessed 20 August 2023). We predict that the *Drosophila CASK*-LOF bushy phenotype will align with in vivo neuronal abnormalities of the human *CASK*-LOF CNS.

The specificity of the bushy neurite-arbor defect make cultured *CASK*-LOF neurons an attractive cell-based assay to study pathogenic mechanisms and to screen for modifying compounds of therapeutic value. Starting with an insect model supports the Three Rs' goal of reducing use of vertebrate research animals [116]. Given the many potential molecular functions of CASK, a cell-based assay would have major advantages over standard medicinal chemistry strategies. First, an unbiased phenotypic screen does not require knowing which protein(s) or signaling pathways should be targeted to improve brain development and function in children with *CASK*-related disorders. Second, recently synthesized CASK inhibitors [133] may have benefit in treating gastrointestinal cancers [33, 126, 158, 167] but would not help *CASK*-LOF neurodevelopmental disorders. In order to efficiently identify enhancers of *CASK*-dependent neurite-arbor morphogenesis, we developed a new method for preparation of primary neuronal cultures.

### A technological breakthrough: overcoming the roadblock of CNS tissue dissociation

We have developed a microfluidic system for automated, standardized, non-fatigable dissociation of brain tissue that yields heathy viable neurons for in vitro cell culture. The arbors elaborated after microfluidic dissociation have excellent quantitative consistency and recapitulate the *CASK*-mutant bushy phenotype (Fig. 8g-j). Moreover, arbors of all genotypes are larger, suggesting the neurons are healthier (Fig. 8a-f). This significant, novel advance follows more than fifty years during which brain-neuron dissociation for primary culture has been an art form, a skill learned at the bench with expert tutelage and much practice. The earliest attempts at separation of individual viable neurons was by extrusion of explanted tissue through a nylon mesh, supplemented by protease treatment and repeated aspiration in media through a Pasteur pipet tip [98, 155, 165]. Decades later, the method still relies on manual trituration of enzyme-treated brain tissue, sometimes using volume-adjustable laboratory pipets, and with the residual-cell-cluster problem being hidden by filtration-based removal [26, 27].

The persistent use of manual dissociation of neural tissues is a testament to the power of primary neuronal culture to advance the understanding of differentiation, synaptic physiology, neurotoxicology, neuropharmacology, and disease pathogenesis [17, 20, 38, 84, 101, 117, 122, 154]. Moreover, several proof-of-concept studies have demonstrated the utility of primary cultured neurons for drug discovery [2, 75, 156, 159]. A compound we identified as an enhancer of the filagree defect of fascin-deficient neurons [75] was validated independently as a fascin-1 inhibitor [4]. Thus, the potential value of our microfluidic system is high for basic and applied neuroscience, and answers an editorial call for more collaboration between geneticists and engineers [1].

Manual neural tissue dissociation has been a 'black box.' The tissue quickly becomes invisible and there is no way to control the applied force, which has to overcome the extracellular matrix and the connections among neurons. Too much trituration reduces neuronal viability, too little leaves cell clusters intact. Even worse, trituration is fatiguing to the dominant hand which limits the number of samples that can be prepared. For CNS tissue from both *Drosophila* and rodents, a controlled bi-directional oscillating flow field through a narrow orifice allows tissue dissociation into viable neurons using cycle frequencies and numbers that would be impossible to generate manually. Moreover, the process can be directly observed in real time (Fig. 7d). The most impactful design improvement was an order of magnitude increase in the length of the orifice in which the dissociation takes place, resulting in a longer time interval during which flow shear stress was exerted on the tissue. The microfluidic system could also dissociate neonatal rat (P1) heart tissue, which tolerated higher stress loads (data not shown).

### Toward high-throughput drug screening using primary neuronal cultures

Due to the increasing availability of massively parallel DNA sequencing in the clinic [161], *CASK*-related and other rare disorders are becoming ever-easier to diagnose [73]. While early molecular diagnosis is of high value, especially when paired with genetic counseling [39], prompt initiation of therapy to enhance brain and cognitive development is an important goal. As with Duchenne/Becker muscular dystrophy [25], one initial strategy would be to convert a severe *CASK* phenotype to a mild one. Together, our discoveries of the *CASK*-LOF bushy neurite-arbor phenotype and an automated microfluidic system for CNS tissue dissociation provide a novel cell-based assay for first-line drug discovery. There are precedents for applying microsystems technology to cell-based assays for drug screening [28, 166] to identify small molecules [152], peptides [114, 163], or antibodies [45, 140]. Compounds that improve the bushy phenotype of mutant *Drosophila* neurons in vitro would then be candidates for testing in other *CASK*-LOF animal models or human neurons from iPSc lines.

*CASK* disorders represent a prime target for drug discovery, not only because of the obvious unmet medical need but also because of CASK is the hub of a genetic regulatory network. These dozen proteins have essential functions in brain and cognitive development, including acquisition of normal brain size (Additional file: Table A1). Drugs that boost CASK function may also be beneficial for disorders caused by mutations in CASK-partner genes.

### Supplementary Information

**Additional file 1: Table A1.** CASK Binding Partners and Target Genes linked to Neurodevelopmental Disease.

**Additional file 2: Table A2.** Case reports and series of CASK-related disorders.

**Additional file 3: Table A3.** Effects of CASK LOF mutation on neurite-arbor morphology: analysis across nine experiments.

**Additional file 4: Figure A1.** Amino acid sequence comparison of human and fruit fly CASK.

**Additional file 5: Figure A2.** Frequency distributions of neurite-arbor parameters of CASK-mutant vs. -control neurons.

**Additional file 6: Figure A3.** Rat E18 hippocampal neurons cultured after dissociation in microfluidic devices.

**Additional file 7: Figure A4.** Consistency of neurite-arbor parameters of neurons dissociated in single- vs. twinchannel microfluidic devices.

## Data Availability

Drosophila stocks used in these studies are available from the corresponding author and/or the Bloomington Drosophila Stock Center. The data generated and analyzed for this research will be made available by the authors upon reasonable request.

## Abbreviations

ASD: Autism spectrum disorder
βgal: β-Galactosidase
CNS: Central nervous system
CRL: Crown-rump length
div: Days in vitro
E/I: Excitatory/inhibitory
FBS: Fetal bovine serum
hESC: Human embryonic stem cells
ID: Intellectual disability
iPSc: Induced pluripotent stem cell(s)
KO: Knockout
LOF: Loss of function
MAGUK: Membrane-associated guanylate kinase
MICPCH: Microcephaly with pontine-cerebellar hypoplasia
PDMS: Polydimethylsiloxane
w3L: Wandering third instar larva(e)
