# Supplementary material for: Drosophila CASK regulates brain size and neuronal morphogenesis, providing a genetic model of postnatal microcephaly suitable for drug discovery
Source: Neural Dev. 2023 Oct 7;18:6. doi: 10.1186/s13064-023-00174-y (PMC10559581; doi:10.1186/s13064-023-00174-y)
Supplement: Supplementary file 1 — Additional file 1: Table A1. CASK Binding Partners and Target Genes linked to Neurodevelopmental Disease. [file 13064_2023_174_MOESM1_ESM.pdf]

Additional Table A1. CASK Binding Partners and Target Genes linked to Neurodevelopmental Disease (Tello et al.)

A. Binding Partners

Protein	Subcellular Localization	Binds CASK domain	Gene	Gene MIM#	NeuroDevelopmental Disorder	Disorder MIM#	Head Size	Transmission, Mechanism	References
BAF chromatin remodeling complex subunit BCL11A	Nucleus	GUK	***BCL11A***	606557	Dias-Logan s.	617101	MiC in some	AD, LOF w/ HI	1, 2, 3
Ca2+ channel, voltage-dependent, N type, alpha-1B subunit	Presynaptic terminal	SH3	***CACNA1B***	601012	NDD w/ Sz and nonepileptic hyperkinetic movements	618497	MiC	AR, predicted LOF	4, 5
Contactin-associated protein-like 2	Soma, dendrites, axons	PDZ	***CNTNAP2***	604569	Pitt-Hopkins like s. 1	610042	MaC in some	AR, probably LOF	6, 7, 8
Cyclin-dependent kinase 5	Presynaptic terminal	CaMK-like, L27	***CDK5***	123831	**Lissencephaly 7 with cerebellar hypoplasia	616342	MiC	AR, LOF	9, 10, 11
FERM domain-containing 7	Plasma membrane	hook b/n SH3 & GUK	***FRMD7***	300628	X-linked congenital nystagmus 1	310700	WNL	XLR or XLD	12, 13, 14
Glutamate receptor-interacting protein 1	Postsynaptic terminal	PDZ	***GRIP1***	604597	*Corpus callosum agenesis, subependymal heterotopia	None	not reported	AR	15, 16
Kirre-like adhesion molecule 3	Pre- & postsynaptic termini	PDZ predicted	***KIRREL3***	607761	IDD, AD 4; *NDD w/ cerebellar hypoplasia	612581; none	low normal	AD	17, 18, 19
Neurexin 1	Presynaptic terminal active zone	PDZ (& other C' regions)	***NRXN1***	600565	Pitt-Hopkins-like s. 2	614325	MiC/low normal	AR	20, 21, 22
Protein Kinase A catalytic subunit B	Soma: membrane, cytoplasm & nucleus	GUK (T724)	***PRKACB***	176892	Cardioacrofacial dysplasia 2	619143	MiC in some	AD	23, 24
Protein 4.1 Neuron Type	Postsynaptic terminal	SH3	***EPB41L1***	602879	*IDD, AD 11	614257	MiC	AD, LOF	25, 26
T-box, brain, 1	Nucleus	GUK	***TBR1***	604616	IDD with autism & speech delay	606053	low normal	AD, LOF w/ HI or DomNeg	27, 28, 29

B. CASK Target Genes

Protein	Subcellular Localization	Binds CASK domain	Gene	Gene MIM#	NeuroDevelopmental Disorder	Disorder MIM#	Head Size	Transmission, Mechanism	References
Reelin	Nucleus	via TBR1	***RELN***	600514	Lissencephaly 2 (Norman-Roberts type)	257320	MiC	AR	30, 31
Glu receptor, ionotropic, NMDA, subunit 2B (NR2b)	Nucleus	via TBR1 and CINAP	***GRIN2B***	138252	DEE 27; IDD, AD 6, +/- Sz	616139; 613970	MiC in some for both	de novo AD, GOF or LOF; AD	32, 33, 34

Abbreviations: *, Single case report; **, Single multiplex family; AD, autosomal dominant; AR, autosomal recessive; b/n, between; DEE, developmental and epileptic encephalopathy; DomNeg, dominant negative; GOF, gain of function; HI, haploinsufficiency; IDD, intellectual developmental disorder; LOF, loss of function; MaC, macrocephaly; MiC, microcephaly; NDD, neurodevelopmental disorder; s., syndrome; Sz, seizures; XLD, X-linked dominant; XLR, X-linked recessive; WNL, within normal limits

References: (1) Dias 2016, PMID 27453576; (2) Kuo 2010, PMID 20623620; (3) Balci 2015, PMID 25979662; (4) Gorman 2019, PMID 30982612; (5) Khanna 2007, PMID 17686036; (6) Gao 2018, PMID 29610457; (7) Gao 2019, PMID 30779956; (8) Strauss 2006, PMID 16571880; (9) Magen 2015, PMID 25560765; (10) Shinmyo 2017, PMID 28854363; (11) Samuels 2007, PMID 18054859; (12) Watkins 2013, PMID 23406872; (13) Betts-Henderson 2010, PMID 19892780; (14) Pu 2019, PMID 31743612; (15) Karaca 2015, PMID 26539891; (16) Hong & Hsueh 2006, PMID 17084383; (17) Ciaccio 2021, PMID 33853164; (18) Taylor 2020 PMID 32503885; (19) Bhalla 2008, PMID 19012874; (20) LaConte 2016, PMID 27015872; (21) Zweier 2009, PMID 19896112; (22) Harrison 2011, PMID 21964664; (23) Palencia-Campos 2020, PMID 33058759; (24) Huang 2010, PMID 20067577; (25) Hamdan 2011, PMID 21376300; (26) Biederer 2001, PMID 11604393; (27) Huang & Hsueh 2017 PMID 28234597; (28) Deriziotis 2014 PMID 25232744; (29) den Hoed 2018 PMID 30250039; (30) Hong 2000, PMID 10973257; (31) Hsueh 2000, PMID 10749215; (32) Wang 2004, PMID 15066269; (33) Platzer 2017 PMID 28377535; (34) Lemke 2014, PMID 24272827
