# Supplementary material for: Drosophila CASK regulates brain size and neuronal morphogenesis, providing a genetic model of postnatal microcephaly suitable for drug discovery
Source: Neural Dev. 2023 Oct 7;18:6. doi: 10.1186/s13064-023-00174-y (PMC10559581; doi:10.1186/s13064-023-00174-y)
Supplement: Supplementary file 2 — Additional file 2: Table A2. Case reports and series of CASK-related disorders. [file 13064_2023_174_MOESM2_ESM.pdf]

Additional File: Table A2. Case reports and series of *CASK*-related disorders (Tello et al.)

First Author[1]	Pub Date	PubMed ID	Number Cases[2]	Unique Cases[3]	Unique Females	Unique Males	Country/Ancestry
Froyen	2007	17546640	1	1	1	0	W. Europe
Hayashi	2008	18629876	1	1	1	0	Japan
Najm	2008	19165920	6	5	4	1	USA, German, Turkish
Piluso	2009	19200522	3	3	0	3	Italian
Tarpey	2009	19377476	12	12	1	11	International study
Hackett	2010	20029458	8	4	1	3	Australia
Moog	2011	21954287	20	20	20	0	Europe, No. African
Burglen	2012	22452838	13	11	9	2	France, Italy
Hayashi	2012	21735175	10	9	9	0	Japan
Saitsu	2012	22709267	2	2	0	2	Japan
Takanashi	2012	23165780	16	6	5	1	Japanese
Valayannopoulos	2012	21609947	3	3	3	0	France
Saleem	2013	23901204	1	1	1	0	"Asian"
Iossifov	2014	25363768	1	1	1	0	'European' ("white")
Michaud	2014	24781210	1	1	1	0	"Asian"
Nakamura	2014	23623288	1	1	0	1	Japan
Moog	2015	25886057	8	8	0	8	W. Europe
Nakajiri	2015	25765806	1	1	1	0	Japan
Wincent	2015	25691404	1	1	1	0	Sweden
Rump	2016	26846091	1	1	1	0	Netherlands
DDDS	2017	28135719	9	9	7	2	multiple
Dunn	2017	28139025	1	1	0	1	USA
Hayashi	2017	28783747	32	16	14	2	Japanese
Muthusamy	2017	28481730	2	2	0	2	authors India, USA
Rivas	2017	28898323	1	1	1	0	Chile
Seto	2017	28944139	2	2	1	1	Japan
Bozarth	2018	30289607	1	1	1	0	USA
Cristofoli	2018	29691940	4	4	4	0	"European"
LaConte	2018	29426960	3	3	3	0	USA, Brazil
LaConte	2019	30549415	1	1	1	0	USA
Murakami	2019	31044082	1	1	1	0	Japanese
Rama Devi	2019	31736593	1	1	0	1	India
Becker	2020	32929080	2	1	0	1	Sweden
González-Roca	2020	32700313	1	1	0	1	Spain
Lee	2020	32247221	1	1	0	1	Korea
Mukherjee	2020	32696595	1	1	0	1	USA
Giacomini	2021	33640666	34	34	29	5	Italy
Nishio	2021	33272775	1	1	1	0	Japan
Pan	2021	33090494	5	5	0	5	multiple
Zhao	2021	33629417	2	2	2	0	Chinese
Ahn	2022	35777792	1	1	1	0	Korean
Dubbs	2022	35670295	11	11	7	4	USA
Khan	2022	35281599	2	2	0	2	Pakistani
Lai	2022	35441233	1	1	1	0	USA
Nuovo	2022	34085948	25	25	21	4	Most Italian
Patel	2022	35149592	1	1	0	1	USA
Tibbe	2022	36137748	4	4	0	4	European?
Wu	2022	35668446	1	1	1	0	Chinese
Xie	2022	36159992	1	1	1	0	Chinese
Yang	2022	36168867	2	2	2	0	Han Chinese
Zhang R	2022	36092876	4	4	2	2	Chinese
Zhang Y	2022	35550617	1	1	0	1	Chinese
**TOTALS**			268	**233**	**160**	**73**	

Female: Male = 2.19

New cases reported after 2020: 96 41.2% of total

Footnotes:

1, within same year, papers listed in alphabetical order; many were e-published the previous year

2, "case" means individuals with clinical neurological phenotype(s) and abnormal *CASK* genotype

3, not previously reported
