# Supplementary material for: Drosophila CASK regulates brain size and neuronal morphogenesis, providing a genetic model of postnatal microcephaly suitable for drug discovery
Source: Neural Dev. 2023 Oct 7;18:6. doi: 10.1186/s13064-023-00174-y (PMC10559581; doi:10.1186/s13064-023-00174-y)
Supplement: Supplementary file 3 — Additional file 3: Table A3. Effects of CASK LOF mutation on neurite-arbor morphology: analysis across nine experiments. [file 13064_2023_174_MOESM3_ESM.pdf]

Additional File: Table A3. (Tello et al.)

Effects of *CASK* LOF mutation on neurite-arbor morphology: analysis across nine experiments

Parameter (units)	Genotype	Mean of the medians	Genotype-mean difference (95% confidence interval)	Standard Error of the Mean	Percent difference, mutant relative to control	*t*-value (df = 8)	p-value (2-tailed *t* test)
**Primary Processes (#)**	*Ex33/Ex33*	5.8	0.1 (-0.6 – 0.8)	0.35	N/A	0.34	0.7397
	Δ18/Δ18	5.9					
**Neurite Length (μm)**	*Ex33/Ex33*	486	168 (107 – 229)	30.5	-35%	-5.18	**0.0008**
	Δ18/Δ18	318					
**Territory Area (μm^2)**	*Ex33/Ex33*	3527	1686 (1074 –– 2298)	306	-48%	-5.75	**0.0004**
	Δ18/Δ18	1841					
**Branch Density (#/1000 μm^2)**	*Ex33/Ex33*	13.3	5.2 (2.98 – 7.6)	1.2	+39%	5.39	**0.0006**
	Δ18/Δ18	18.5					
**Higher-Order Branches (#)**	*Ex33/Ex33*	37	11 (6 – 16)	2.5	-30%	-4.39	**0.0023**
	Δ18/Δ18	26					
**Branches (#)**	*Ex33/Ex33*	43.5	11.2 (6.0 – 16.4)	2.6	-26%	-3.9	**0.0045**
	Δ18/Δ18	32.3					
**Branches/Length (#/100 μm)**	*Ex33/Ex33*	9.1	1.2 (0.62 – 1.78)	0.29	+13%	4.63	**0.0017**
	Δ18/Δ18	10.2					
**HO Branch Density (#/1000 μm^2)**	*Ex33/Ex33*	11.2	3.4 (1.32 – 5.28)	0.94	+30%	4.34	**0.0025**
	Δ18/Δ18	14.8					
