# Supplementary material for: Drosophila CASK regulates brain size and neuronal morphogenesis, providing a genetic model of postnatal microcephaly suitable for drug discovery
Source: Neural Dev. 2023 Oct 7;18:6. doi: 10.1186/s13064-023-00174-y (PMC10559581; doi:10.1186/s13064-023-00174-y)
Supplement: Supplementary file 4 — Additional file 4: Figure A1. Amino acid sequence comparison of human and fruit fly CASK. [file 13064_2023_174_MOESM4_ESM.pdf]

Tello et al.

Additional File: Figure A1.

Amino acid sequence comparison of human and fruit fly CASK.

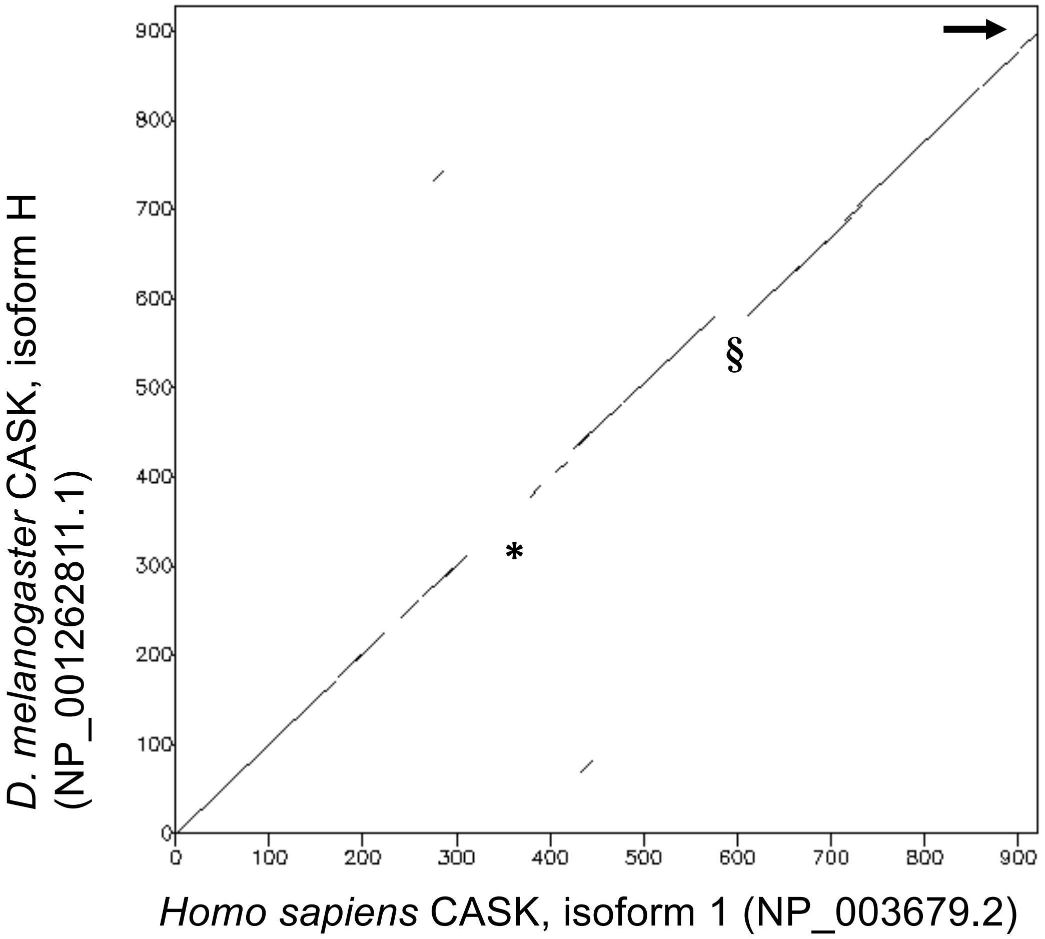

Dot-matrix representation of the amino acid sequence similarities of human and fruit fly CASK. Extensive regions of amino acid conservation are indicated by the diagonal line segments. The gap (asterisk) is a region of lower similarity. The human sequence has a 35-amino acid insertion (§) relative to the fly sequence, seen as a lateral shift to the right in the diagonal line position. At the end of the sequences, the C-terminal tail is shorter in the fly protein than in the human ortholog (arrow).
