# Supplementary material for: Drosophila CASK regulates brain size and neuronal morphogenesis, providing a genetic model of postnatal microcephaly suitable for drug discovery
Source: Neural Dev. 2023 Oct 7;18:6. doi: 10.1186/s13064-023-00174-y (PMC10559581; doi:10.1186/s13064-023-00174-y)
Supplement: Supplementary file 5 — Additional file 5: Figure A2. Frequency distributions of neurite-arbor parameters of CASK-mutant vs. -control neurons. [file 13064_2023_174_MOESM5_ESM.pdf]

Tello et al.

Additional File: Figure A2.

Frequency distributions of neurite-arbor parameters of *CASK*-mutant vs. -control neurons

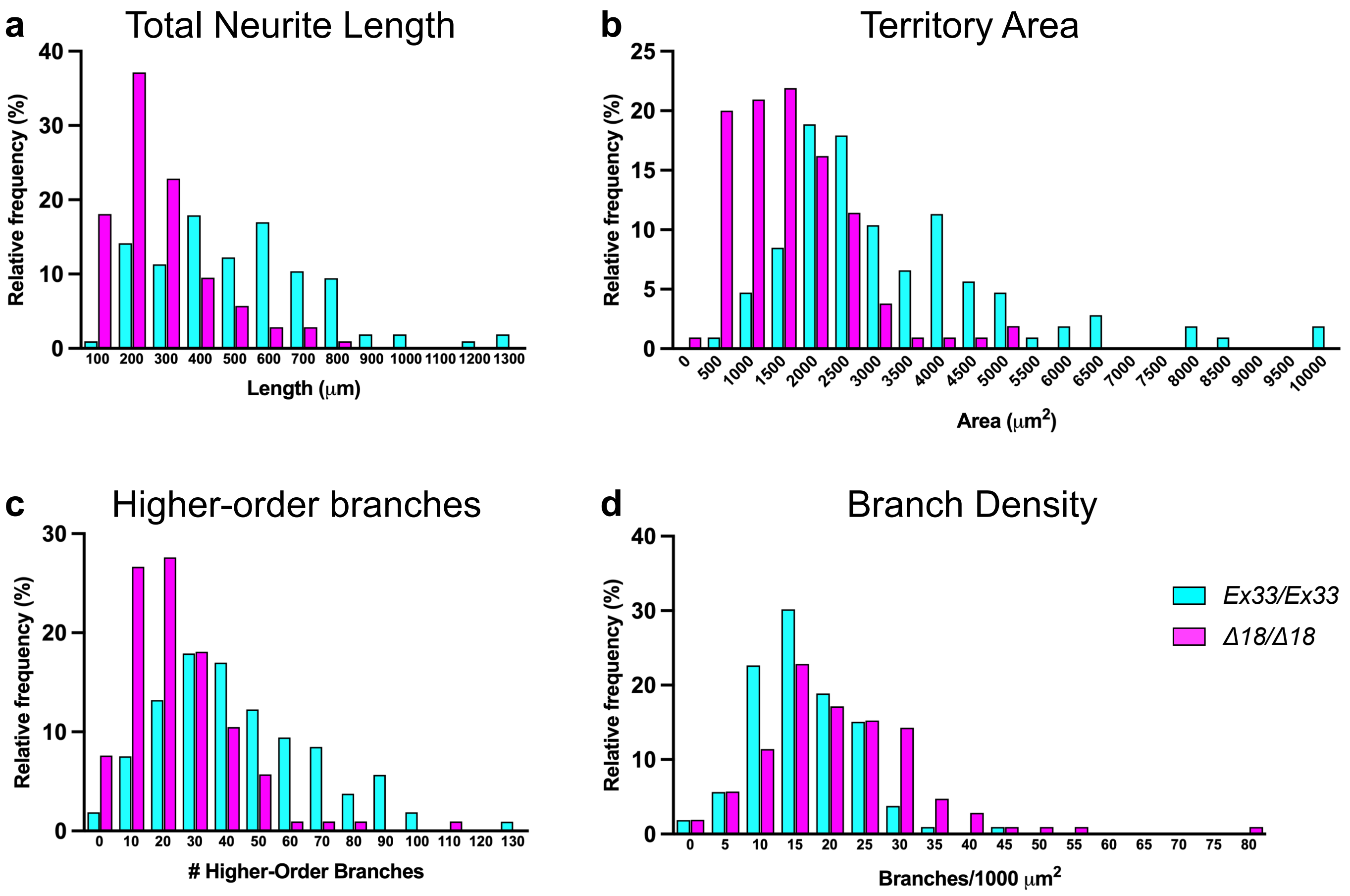

Histograms depicting the distribution of neurite-arbor parameters of *CASK*-mutant (Δ*18/*Δ*18*) and *CASK*-control (*Ex33/Ex33*) cultured neurons. Data from the experiment shown in Figure 3 of the main paper. Note that many of the data sets are not normally distributed. The mutant neurons have reduced (a) total neurite length, (b) territory area, and (c) higher-order branch number, but increased (d) branch density, compared with control neurons. While very skewed, the distributions of mutant-neuron parameter values are unimodal. There is no obvious indication of a subset of mutant neurons that is unaffected by *CASK* LOF.
