# Supplementary material for: Drosophila CASK regulates brain size and neuronal morphogenesis, providing a genetic model of postnatal microcephaly suitable for drug discovery
Source: Neural Dev. 2023 Oct 7;18:6. doi: 10.1186/s13064-023-00174-y (PMC10559581; doi:10.1186/s13064-023-00174-y)
Supplement: Supplementary file 6 — Additional file 6: Figure A3. Rat E18 hippocampal neurons cultured after dissociation in microfluidic devices. [file 13064_2023_174_MOESM6_ESM.pdf]

Tello et al.
Additional File: Figure A3.
Rat E18 hippocampal neurons cultured after dissociation in microfluidic devices.

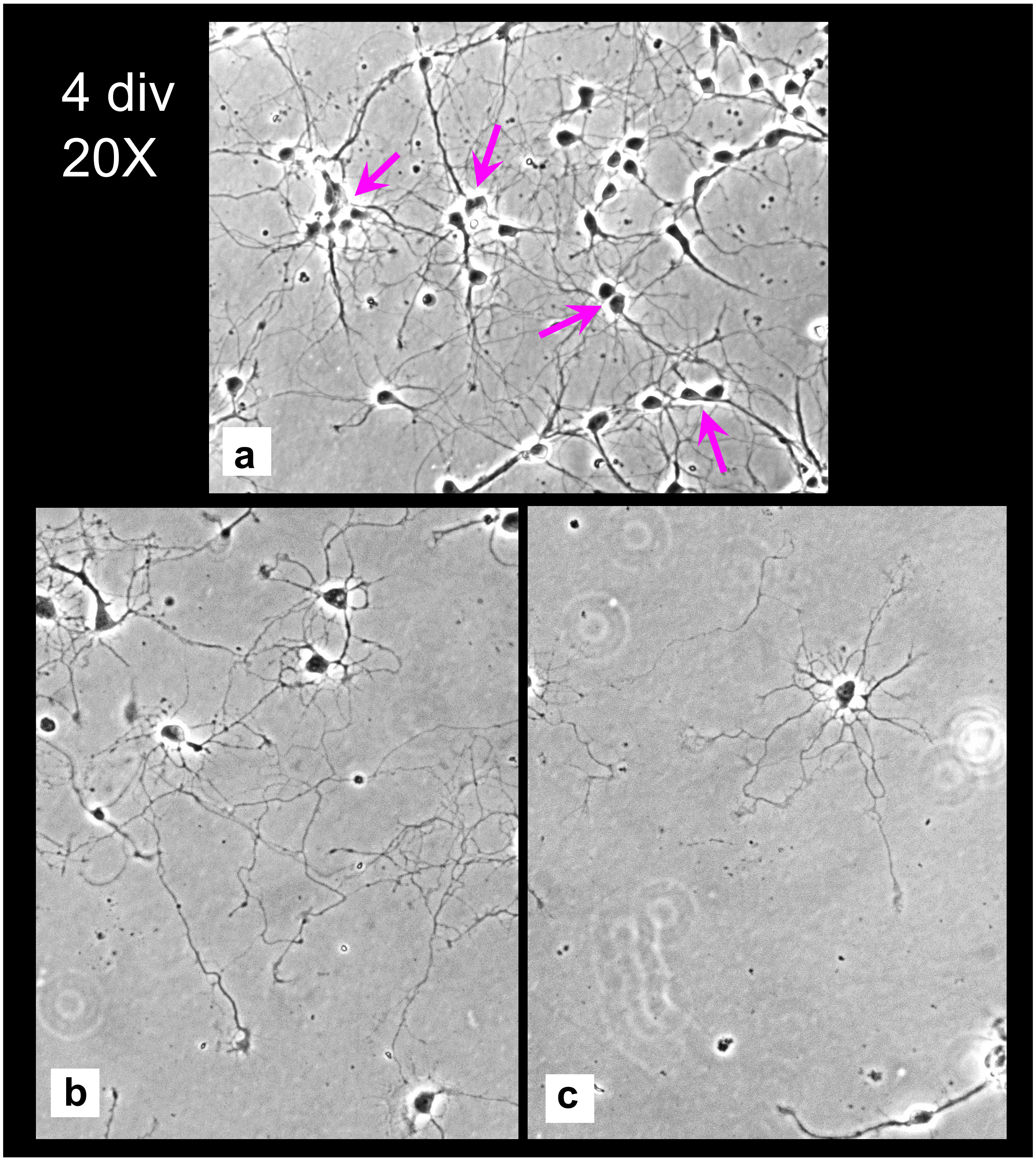

Photomicrographs of 4 div neuronal cultures after dissociation of hippocampal tissue performed in microfluidic devices with the following dimensions: height 500 μm; orifice length 400 μm; orifice width 70 μm. Flow parameters were infusion volume 12.5 μL; flow rate 50 μL/sec; oscillation frequency 4 Hz. (a) With relatively low cycle number (360), the dissociation was incomplete. Although there were no tissue clumps remaining, there were many small cell clusters (arrows) as seen in this relatively high-density culture. Nonetheless, neurite outgrowth was robust. (b) In subsequent experiments with increased cycle number (1,444), there was greater likelihood of complete dissociation into single neurons. (c) When the neurons were plated at low density, individual neurite arbors of varying complexity and morphology could be seen.
