# Supplementary material for: Drosophila CASK regulates brain size and neuronal morphogenesis, providing a genetic model of postnatal microcephaly suitable for drug discovery
Source: Neural Dev. 2023 Oct 7;18:6. doi: 10.1186/s13064-023-00174-y (PMC10559581; doi:10.1186/s13064-023-00174-y)
Supplement: Supplementary file 7 — Additional file 7: Figure A4. Consistency of neurite-arbor parameters of neurons dissociated in single- vs. twinchannel microfluidic devices. [file 13064_2023_174_MOESM7_ESM.pdf]

Tello et al.

Additional File: Figure A4

Consistency of neurite-arbor parameters of neurons dissociated in single- vs. twin-channel microfluidic devices.

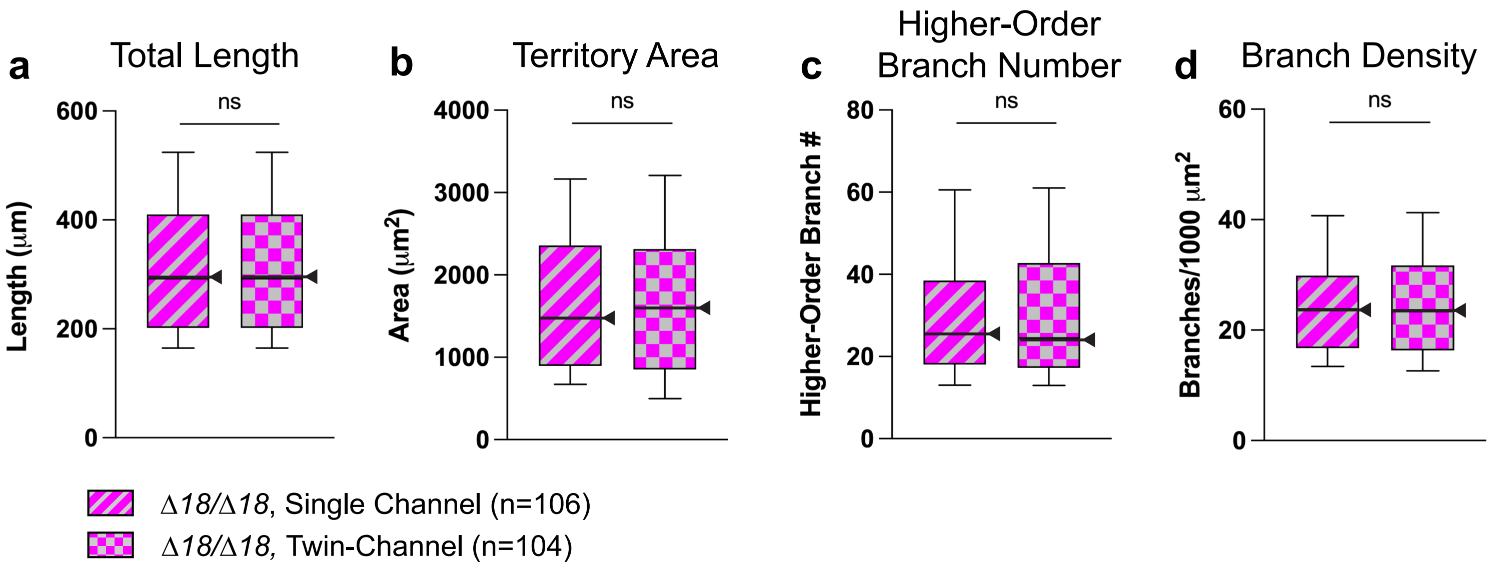

Neurite-arbor size and shape quantification, comparing neurons dissociated from two Drosophila w3L CNS of the same genotype (*CASK*-mutant Δ*18*/Δ*18*) and cultured for 3 div. The two samples were prepared in series during the same experimental session and dissociated in either a single-channel or a twin-channel device. Flow parameters were infusion volume 10.5 µL; flow rate 50 µL/sec; oscillation frequency 4.8 Hz. All reagents, such as enzymes, culture media, and dish coating, were identical. Note the remarkable consistency of the size-and-shape parameters of neurite arbors elaborated in this pair of neuronal cultures. Sample size and pattern legend as indicated. ns, not significant
